# Economic Impact of HIV and Antiretroviral Therapy on Education Supply in High Prevalence Regions

**DOI:** 10.1371/journal.pone.0042909

**Published:** 2012-11-16

**Authors:** Claire L. Risley, Lesley J. Drake, Donald A. P. Bundy

**Affiliations:** 1 The Partnership for Child Development, Imperial College, London, United Kingdom; 2 Liverpool University Climate and Infectious Diseases of Animals (LUCINDA) Group, Institute of Infection and Global Health, University of Liverpool, Neston, Cheshire, United Kingdom; 3 Human Development Network, The World Bank, Washington DC, United States of America; Northeastern University, United States of America

## Abstract

**Background:**

We set out to estimate, for the three geographical regions with the highest HIV prevalence, (sub-Saharan Africa [SSA], the Caribbean and the Greater Mekong sub-region of East Asia), the human resource and economic impact of HIV on the supply of education from 2008 to 2015, the target date for the achievement of Education For All (EFA), contrasting the continuation of access to care, support and Antiretroviral therapy (ART) to the scenario of universal access.

**Methodology/Principal Findings:**

A costed mathematical model of the impact of HIV and ART on teacher recruitment, mortality and absenteeism (Ed-SIDA) was run using best available data for 58 countries, and results aggregated by region. It was estimated that (1) The impact of HIV on teacher supply is sufficient to derail efforts to achieve EFA in several countries and universal access can mitigate this. (2) In SSA, the 2008 costs to education of HIV were about half of those estimated in 2002. Providing universal access for teachers in SSA is cost-effective on education returns alone and provides a return of $3.99 on the dollar. (3) The impacts on education in the hyperendemic countries in Southern Africa will continue to increase to 2015 from its 2008 level, already the highest in the world. (4) If treatment roll-out is successful, numbers of HIV positive teachers are set to increase in all the regions studied.

**Conclusions/Significance:**

The return on investing in care and support is also greater in those areas with highest impact. SSA requires increased investment in teacher support, testing and particularly ART if it is to achieve EFA. The situation for teachers in the Caribbean and East Asia is similar but on a smaller scale proportionate to the lower levels of infection and greater existing access to care and support.

## Introduction

### Background to study

The global economic model framework used by this research was originally commissioned by the UNAIDS/World Bank Economic Reference Group (ERG) for presentation of initial results at its 2007 meeting “The Impact of HIV and AIDS on Educational Attainment and Human Capital”, UNAIDS headquarters, Geneva. This paper is hosted on the Partnership for Child Development website (http://www.schoolsandhealth.org/Documents/Estimating%20the%20impact%20of%20HIV%20and%20AIDS%20on%20the%20supply%20of%20basic%20education.pdf)

### HIV and the teacher workforce

Teachers are instrumental in the achievement of Education For All and the second Millennium Development goal (MDG) of universal access to quality basic education [1]. Teachers are also an important share of the salaried employee base in most countries, often constituting some 50–60% of the public sector workforce, with a salary bill that represents between 60% and 98% of the budget of ministries of education [2] Teachers also occupy a special place in combating the epidemic as they are key agents of change for HIV prevention among youth (see for example [3],[4]). All of which suggests that, in addition to the human dimension, maintaining the current and future supply of teachers despite the HIV epidemic is critical to education quality, economic stability and the future control of the epidemic.

### Previous estimates of the economic cost of HIV to the education sector

Given the scale of the teacher population and the importance of education, there have been surprisingly few previous attempts to estimate the incremental costs of HIV & AIDS for achieving EFA, and only two which have sought to provide a regional perspective. Both of these are for sub-Saharan Africa and both were first published in the 2002 EFA Global Monitoring Report [5], though one was published subsequently in a different form by Bruns, Mingat and Rakotomalala in 2003 [6]. These two early studies both attempted a comprehensive estimate of costs, including the costs of prevention programs and support for affected children. The EFA Global Monitoring Report estimate is $975million per year, of which $300 million addresses the supply side issues, replacement of teachers ($150 million) and temporary teachers ($150 million). The methodology for deriving these estimates is not presented, nor the specific countries covered. The Bruns et al. estimate [6] is $287 million per year for 33 countries in sub-Saharan Africa, including those worst affected by HIV & AIDS. This estimate includes supply side costs and also an element for support of orphans. The analysis was based on an economic model and it is not possible, based on the published data, to separate the supply from the demand elements. The total cost of this estimate however is of similar order of magnitude to the supply side cost estimate in the EFA Global Monitoring Report. Studies are beginning to address the importance of this issue; notably the study in to ART takeup of teachers in Malawi [7].

### Results are presented regionally and globally

Given the geographical variation in HIV prevalence [8], we took a regional approach. We estimated the economic impact of HIV on teaching supply in the three geographical regions estimated by UNAIDS to be the worst affected by the pandemic: sub-Saharan Africa, the Caribbean and the Greater Mekong sub-Region of East Asia (see Table S1 for specific countries). All of these regions include countries where the epidemic prevalence exceeds 1% and is defined as generalized. UNAIDS/WHO. Second generation surveillance guidelines. Geneva:UNAIDS/WHO, 2000.

HIV is most prevalent regionally in sub-Saharan Africa. Of the 44 sub-Saharan African countries evaluated by UNAIDS, 38 (86%) are experiencing a generalized epidemic, and only Madagascar, Mauritius, Mauritania, Senegal, Somalia and the Comoros are estimated to have an HIV prevalence of less than 1% [8]. Never-the-less there is very considerable variation within the region, so here we group the countries into four sub-regions (Table S1). Prevalence is peaking in southern Africa, where the impact has been greatest [9], whereas in the other three sub-regions of the continent prevalence peaked in the 1990's and is in decline (figure 11.9 of [8]). Since, where ART uptake is low, peaks in AIDS mortality follow peaks in HIV incidence by approximately 11 years (the average time from infection until death [10]) the impact of HIV is probably now declining in much of sub-Saharan Africa, with the exception of Southern Africa where the greatest impact is yet to be realized.

The Caribbean is estimated to be the second most affected region in the world, with seventeen (63%) out of 21 Caribbean countries experiencing a generalized epidemic [11], [12]. In Asia, the Greater Mekong sub-Region (East Asia) has an unusually high regional prevalence, with generalized epidemics in three of the five countries [8].

### Modelling approach

As AIDS mortality and absenteeism increased in the nineties and beyond, tools were developed to help countries respond, including Ed-SIDA [13], [14], [15] which has been used locally in some 24 countries to project the impact of HIV on EFA planning profiles for teacher recruitment and for an economic analysis [16]. This model dovetails with the epidemiological outputs of UNAIDS' EPP and Spectrum, which are used to model the age-gender profile of HIV infection and AIDS mortality. This age-gender profile is then applied to data on the age-gender profile of teachers to estimate their risk of infection. This model has been fully updated from earlier versions for the present analysis; in the age of ART, models must incorporate their mitigating effect, and in its latest (2009) version [15] Ed-SIDA allows the user to vary future VCT and ART uptake to estimate its impact.

Our data are from a variety of sources and the data collection effort associated with this study brought together these health, education and economic data for the first time here. UN databases were the source of the bulk of the data, particularly UIS and WHO databases. Teacher age distributions, attrition, recruitment, salary, training costs and death benefits were sourced from the published literature and directly from government institutions (primarily Ministries of Education) in partner countries. Data were sourced directly from Eritrea, Kenya, Nigeria, Guyana, Trinidad & Tobago and Jamaica, detailed in Tables S2 and S3.

In this paper, we build on previous analyses, using these latest methods and data to examine quantitative aspects of the impact of HIV on education supply, estimating numbers of teachers dying due to AIDS and teachers absent due to AIDS illnesses, and also accounting for the financial impact of HIV on education. The impact of HIV on the demand for education is an issue of considerable importance, especially through its effects on orphans and vulnerable children who may have less access to schooling (see for example http://www.unicef.org/infobycountry/), and is being addressed in a separate analysis.

To estimate the impact of HIV on EFA achievement, we use the enrolment and teacher numbers, in addition to existing teacher recruitment and attrition rates, to see how countries must increase their efforts in order to achieve EFA. We then compare this with the counterfactual scenario without the deaths of teachers due to the HIV epidemic. the impact that the epidemic has had on the ease of EFA achievement, and apply ART scenarios to 2015 to assess the effect that treatment could have on EFA achievement.

### Overall Approach

The study described here explores, for three regions with generalized HIV epidemics, the impact of HIV on teacher supply in basic education in 2008 to 2015, the target date for the achievement of EFA. The study estimates the additional economic costs due to HIV of providing sufficient teachers to achieve EFA and the education MDG, and projects the costs under two scenarios reflecting different levels of availability of care and support, including Antiretroviral therapy, to teachers.

Following extensive data collection, analyses were performed for each of a total of 53 countries in three regions: sub-Saharan Africa (separated into four geographical sub-regions; West, Central, East and Southern), the Caribbean and the Greater Mekong sub-region of South East Asia. The country results were then summed to provide an aggregate regional or sub-regional total. For the basic education sector in each region, we estimate and project to 2015 the following impacts due to HIV: 1) The number of teachers made vulnerable due to HIV infection or needing care and support; 2) the numbers of teachers dying and time spent absent due to illness, 3) the consequences of these human resource impacts on education supply, 4) the costs to the education sector of this reduction in education supply in the context of EFA achievement, and 5) the costs and benefits of increasing teacher access to VCT and ART. The sensitivity of the results to important parameters was then evaluated.

## Results

### Data collected

**Figure 1:**
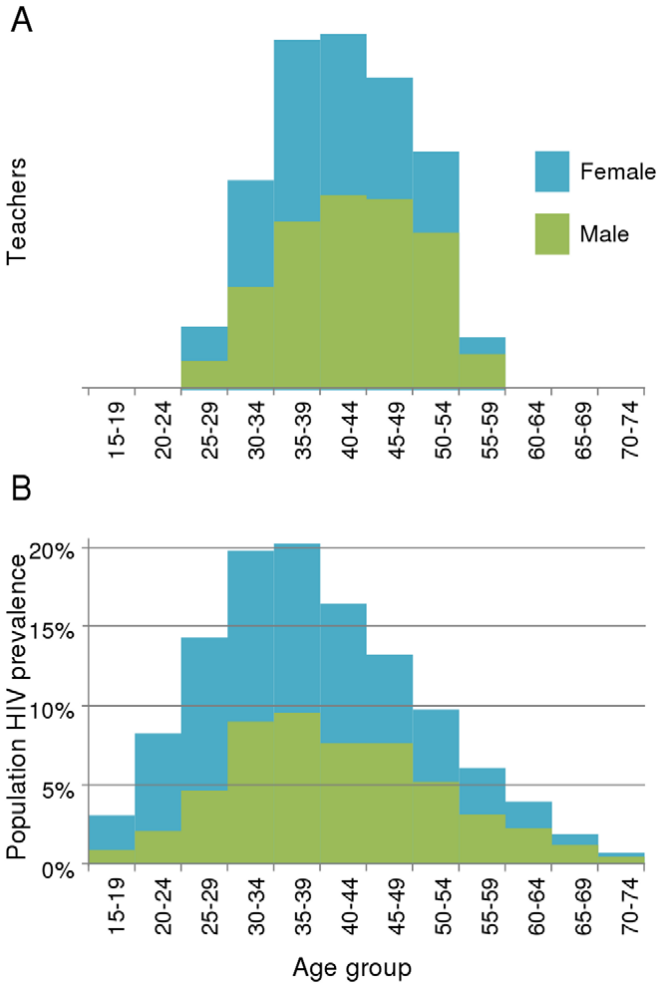
Teacher susceptibility to HIV due to age and gender. A. Age distribution of teachers employed in Kenya, 2005, by gender (data source: Kenya Teachers Service Commission), input into the model for each country in the East Africa sub-region. B. Age distribution HIV infection in sub-Saharan Africa, 2005, by gender, calculated using EPP and spectrum.

We report here demographic data on teachers across continents, some of which is new (asterisked in Tables S2 and S3), and the rest of which has been gathered from a broad array of sources, including DHS surveys, the SACMEQ surveys, individual studies in the academic and grey literature in addition to UNESCO education statistics. These teacher data can be compared with HIV demography by region to comment on teacher demographic risk of infection. Teachers in basic education in sub-Saharan Africa (SSA) are mostly male, whereas in the Caribbean, female teachers predominate. The situation in East Asia is mixed, with Myanmar and Vietnam having mostly female teachers and the other three countries having approximately equal numbers (see Table S2). In most countries in SSA, more women than men are infected with HIV; whereas the opposite is true of many countries in Asia and the Caribbean. The teacher gender profile in most countries thereby biases prevalence lower relative to the working population. By contrast to the teacher gender distribution, the working population as a whole tends to belong to age groups more susceptible to HIV, and teachers are not an exception (figure 1). According to our results, the inflating effect of the age–bias exceeds the deflating effect of gender-bias, meaning that the age-gender groups teachers belong to have an HIV prevalence higher than the general population.

All except five of the countries investigated in sub-Saharan Africa had a generalized epidemic, with a prevalence of >1%, with much the highest prevalence in Southern Africa where some countries have prevalences in excess of 20%. In the Caribbean, all countries investigated were experiencing generalized epidemics with prevalence >1%, but with a smaller range – the highest prevalences (in Haiti and the Bahamas) are less than 2%. Of the East Asian countries, Thailand is experiencing a generalized epidemic of greater than 5%, and Cambodia is just below at 0.8% prevalence [8].

From UNAIDS estimates of ART access in 2007, only 6 countries in sub-Saharan Africa exceed 50% coverage of those in need, four of which are in one sub-region (Southern Africa). In the Caribbean, all countries exceed 50% coverage with the exceptions of the Dominican Republic, Haiti and Jamaica. In East Asia, 3 countries exceed 50% coverage and only Vietnam and Myanmar do not.

**Figure 2:**
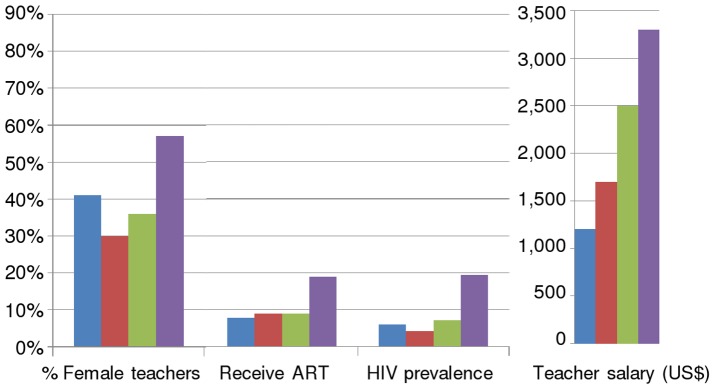
Selected model input data for each region in sub-Saharan Africa, 2007. Female teachers are presented as a percentage of all teachers; Receive ART is presented as a percentage of the population needing ART; Prevalence is the population HIV prevalence [8] ; salary is in US$.

Data inputs are presented for each region within sub-Saharan Africa in figure 2:

### Model outputs

#### 1. Estimating the number of teachers living with HIV

We firstly estimated the total number of teachers who currently require care and support because they are HIV positive. We found a total of 265,000 teachers living with HIV in the countries investigated. The large majority of these live in sub-Saharan Africa (254,000), distributed sub-regionally among: West Africa (47,000), Central Africa (26,000), East Africa (69,000) and Southern Africa (111,000). There are substantially fewer in the Caribbean Region (2,000), and in East Asia (9,000).

**Figure 3:**
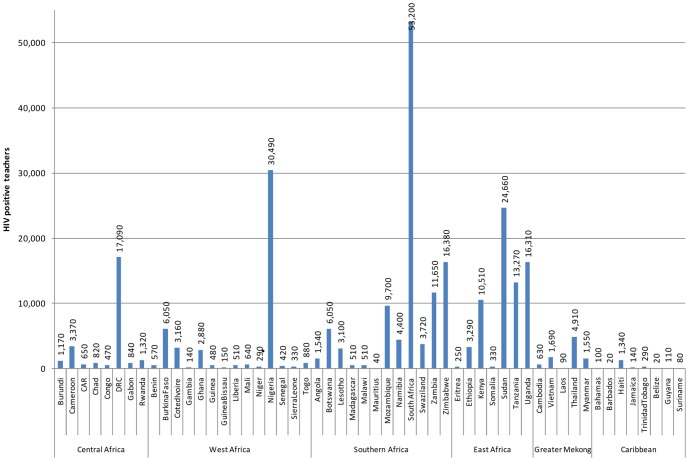
HIV positive teachers in 2009 by country estimated by this analysis, rounded to the nearest 10.

Figure 3 displays numbers of HIV positive teachers by country. The countries estimated to have the most HIV positive teachers are South Africa, Nigeria, Democratic Republic of Congo, and Sudan.

In the following three figures, we look at impacts of HIV on teachers in each region to 2015, and from 2009, compare scenario (1), ART at status quo, with scenario (2), immediate universal access, and error bars display the range of the uncertainty in the underlying severity of the HIV epidemic.

**Figure 4:**
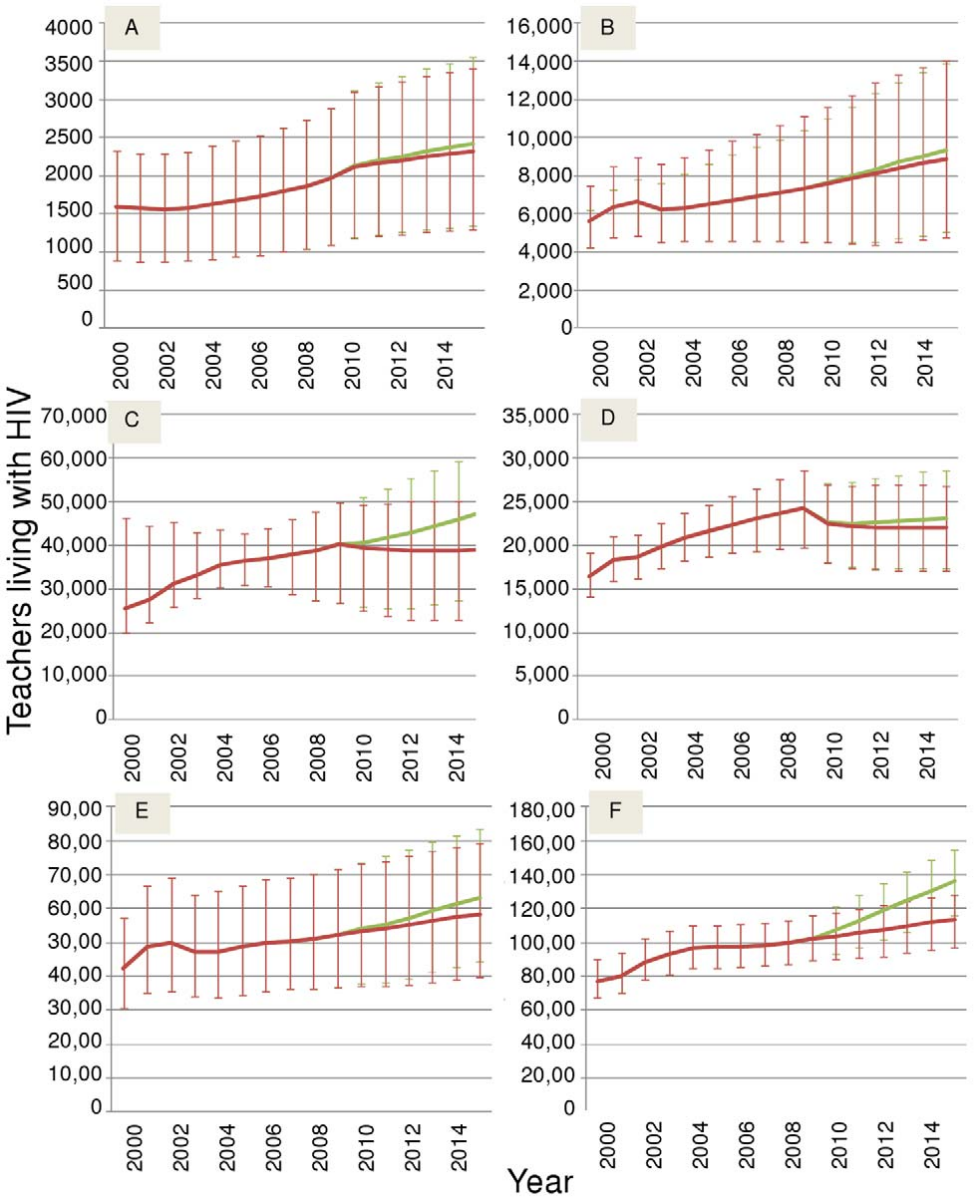
Estimated and projected HIV positive teachers and the future impact of increased ART take-up on their numbers. The red line (on top) indicates treatment remaining at 2008 levels; the green line (beneath) represents treatment increase to a maximum level. A: Caribbean; B: East Asia; C: West Africa; D: Central Africa; E: East Africa; F: Southern Africa 2000–2015. Error bars show the uncertainty due to uncertainty in the population HIV estimates. Teacher recruitment is at current levels.

Numbers of teachers living with HIV (figure 4) are most likely to increase, especially if there is universal access to ART; likely increases in TLWH are apparent everywhere as ART access is increased, and also as it is kept at status quo in the Caribbean, East Asia, and Eastern and Southern Africa (in West and central Africa, numbers of HIV positive teachers are projected to remain steady under status quo as infection and TLWH recruitment balance AIDS deaths). Although this increase is most likely, the effect of the uncertainty in epidemic severity means that we are not certain of this result, that is, error bars also allow flat trajectories. The only place in which an unequivocal increase in the number of teachers living with HIV is expected under all treatment and epidemic scenarios (uncertainty bars do not cross) is Southern Africa.

These are the best estimates given the data available. These figures are subject to error due to epidemic scenario, teacher age-gender profile, and teacher relative risk, but as these are regionally summed estimates, it is most likely that this variation will not sum to bias the estimates one way or another (See scenario analyses section below).

#### 2. Projecting the changes in the impact of HIV on absenteeism and deaths

**Figure 5:**
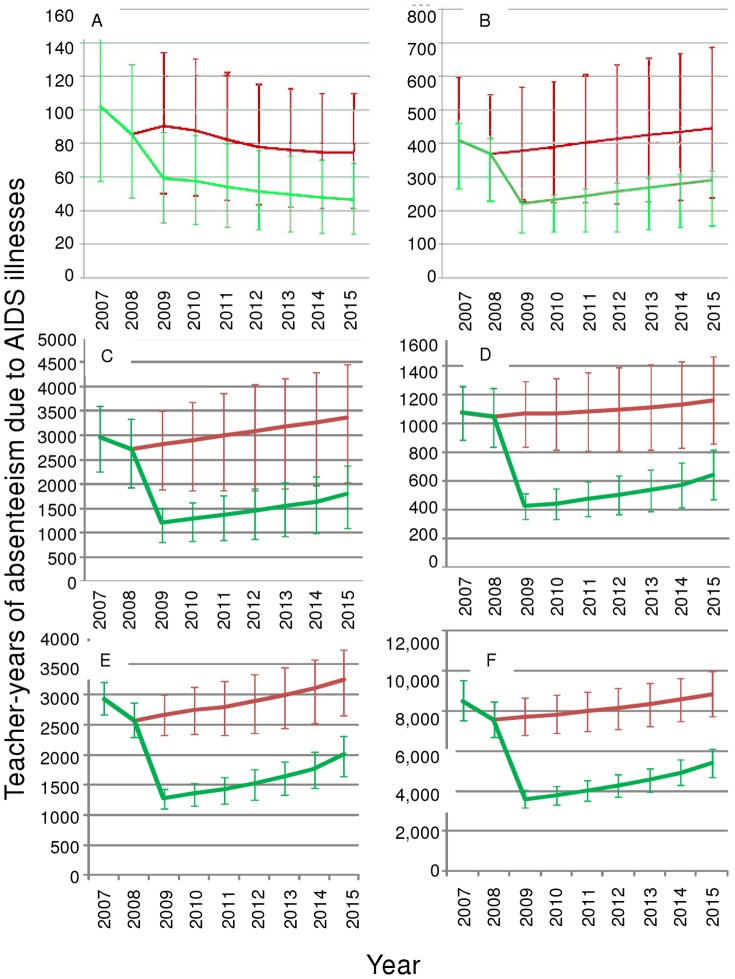
Estimated and Projected Teacher AIDS absenteeism and the future impact of increased ART access. The red line (on top) indicates treatment remaining at 2008 levels; the green line (beneath) represents treatment increase to a maximum level. A: Caribbean; B: East Asia; C: West Africa; D: Central Africa; E: East Africa; F: Southern Africa 2007–2015. Error bars show the uncertainty due to uncertainty in the population HIV estimates. Teacher recruitment is at current levels.

**Figure 6:**
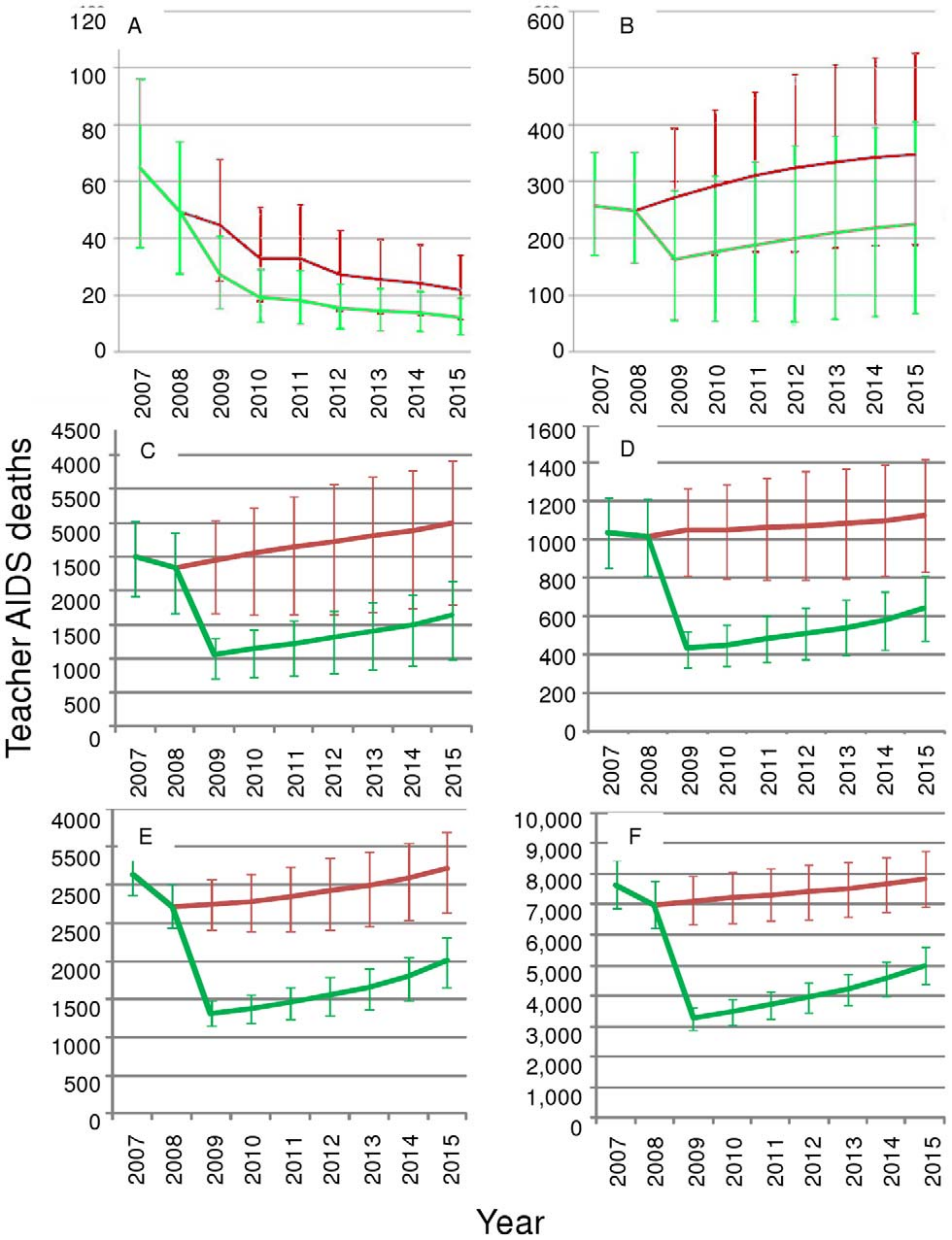
Estimated and projected teacher AIDS deaths. The red line (on top) indicates treatment remaining at 2008 levels; the green line (beneath) represents treatment increase to a maximum level. A: Caribbean; B: East Asia; C: West Africa; D: Central Africa; E: East Africa; F: Southern Africa 2007–2015. Error bars show the uncertainty due to uncertainty in the population HIV estimates. Teacher recruitment is at current levels.

Next we looked at impacts through time of HIV and ART on education supply, estimating the number of teachers dying from AIDS and teacher-years of absenteeism (Figures 5 and 6) If treatment is maintained at current levels (red lines), both absenteeism and deaths are likely to decrease in the Caribbean (Panel A, figures 5 and 6). In East Asia and all sub-regions of SSA, maintaining historical levels will lead to an increase in absences and deaths(red lines figure 5 and 6 panels B–F), indicating the inadequacy of 2008 ART rollout.

These trends among teachers under the full treatment scenario (green lines) are true under most high and low epidemic uncertainty scenarios; the exception is the lower scenario of teacher deaths in East Asia, where stabilization becomes more likely than an increase (figure 6B). Comparing across regions, this uncertainty in the epidemiological model indicates that we are much more certain that ART will significantly influence absenteeism and deaths in central, East and Southern Africa than elsewhere; in these areas the uncertainty bars around the lines do not overlap.

The immediate and sustained provision of ART could reduce AIDS absenteeism by 40%, and teacher AIDS deaths by 38% globally.

We project that in the absence of ART increases, a year-on-year increase in teacher AIDS mortality from current levels is expected in Central, Southern and Western Africa, and East Asia from now to 2015 (output not shown). In all areas, HIV prevalence is expected to increase. Increasing treatment globally reduces the proportion of teachers dying by 39% (from 0.45%–0.28%) and increases proportion of teachers infected with HIV by 19% (from 7.7%–9.1%)

#### 3. Overall impact of HIV on teachers across regions

The impacts of HIV on teachers can be compared between regions by examining HIV prevalence and AIDS mortality rate (table 1). Southern Africa has by far the largest prevalence and mortality; and sub-Saharan Africa as a whole, is experiencing a greater impact than the other global regions.

**Table 1:** Estimated teacher HIV prevalence and AIDS mortality rate, six world regions, 2008 Source: This analysis.

Geographic Region	HIV prevalence in teachers	AIDS mortality in teachers
West Africa	4.2%	0.23%
Central Africa	4.8%	0.33%
East Africa	7.1%	0.44%
Southern Africa	20.3%	1.22%
Caribbean	2.6%	0.07%
East Asia	0.9%	0.03%

#### 4. The impact of HIV on pupil-teacher ratios and necessary teacher recruitment in sub-Saharan Africa

A high pupil-teacher ratio (PTR) in a country indicates that on average, class sizes are larger than ideal, and the average quality of education may consequently be inferior to a country with a lower PTR. The Fast Track Initiative's commonly used target for a PTR above which education quality is inadequate is 40. The achievement of EFA therefore depends on recruitment and retainment of sufficient teachers before 2015 to achieve or maintain a PTR below 40, even if all children of school age are enrolled in school (i.e. if net enrolment rate is increased to 100%). In this section we refer only to the most likely epidemic scenario (not high and low) as the results are not qualitatively different.

The analysis of required recruitment summarized in Table 2 shows Central and East Africa to be the regions of most concern within SSA; with respectively 5- and 3.9-fold increases in recruitment necessary. Even West Africa, with a current PTR of 41, requires a doubling of recruitment effort to achieve a PTR of 40 in every country (due to the enrollment increases also required there- see below).

**Table 2:** Recruitment required for EFA achievement and 2008 teachers, pupil-teacher ratios and annual recruitment in sub-Saharan African regions.

African Region	West	Central	East	Southern	sub-Saharan Africa
Teachers in 2008 (1,000's)	996	311	618	569	2474
Children in school in 2008 (1,000's)	40760	16915	36524	24987	118824
Pupil-teacher ratio	41	54	59	44	48
Baseline recruitment (1,000's)	77	15	46	35	172
Annual recruitment required to achieve EFA (if enrolment is also increased; 1,000's)	178	75	178	89	519
-fold increase required	2.3	5	3.9	2.6	3

For each sub-Saharan African sub-region in turn, below we detail the increases in teachers and teacher recruitment required to achieve EFA, the impact on this that treating teachers would have and give an indication of the power of HIV prevention by reporting what effect HIV infection had on required recruitment.

**Figure 7:**
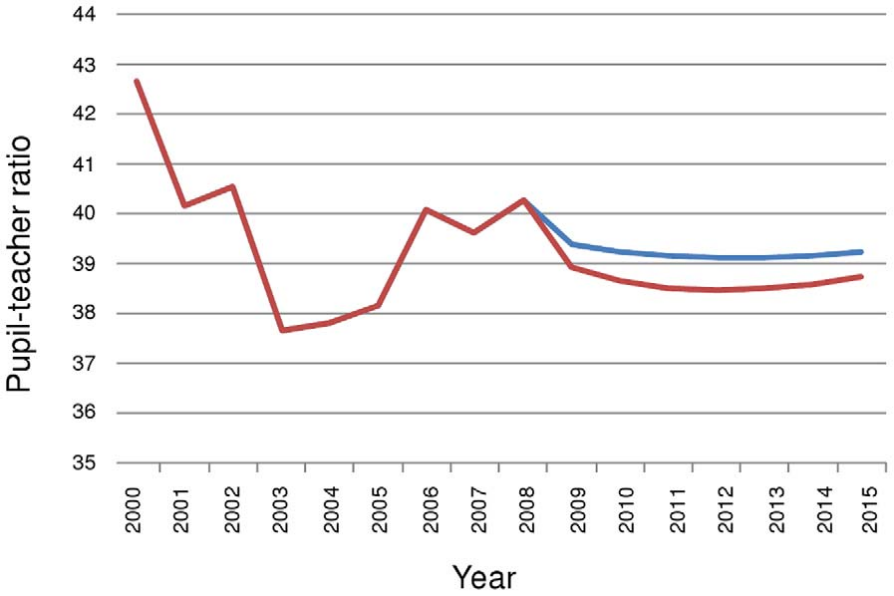
Estimated and projected pupil-teacher ratio in West Africa 2000–2015. The blue line indicates recruitment at current levels; the red line where current rates of recruitment are increased such that EFA is achieved in each country.

West Africa as a whole (Figure 7) in addition to most West African countries (data not shown) are projected to have achieved a pupil-teacher ratio of less than 40 by 2015 with current rates of enrolment and teacher recruitment. However, West African countries tend to have lower enrolment in addition to lower pupil-teacher ratios than countries in other SSA regions. The extra pupils required to increase enrolment to 100% by 2015 would mean that extra teacher recruitment efforts would be required in most West African countries to achieve the EFA target. In the case of increased enrolment, only Senegal, Liberia and Ghana are set to achieve a pupil-teacher ratio less than 40 in 2015.

**Figure 8:**
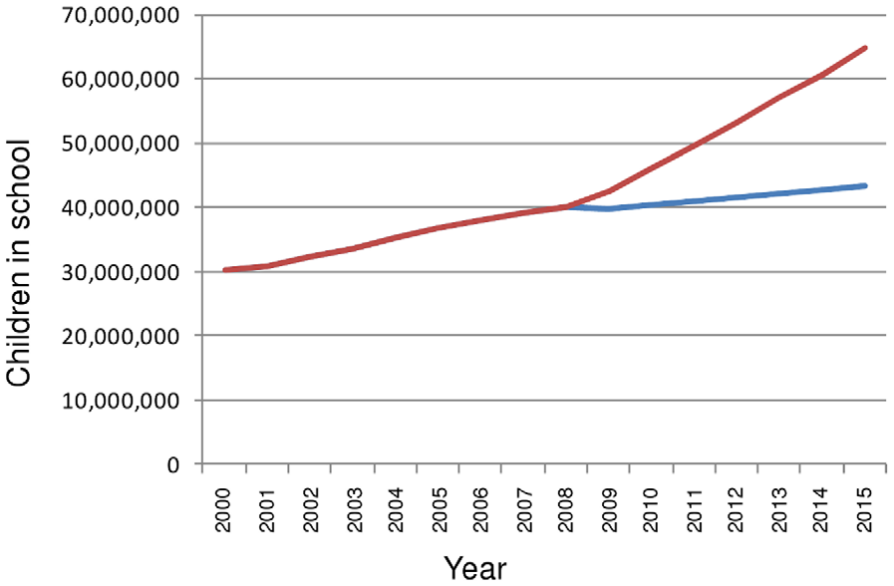
Estimated and potential numbers of Children in school in West Africa 2000–2015. The blue line indicates recruitment at current levels; the red line where current rates of recruitment are increased such that EFA is achieved in each country.

As enrolment rates are currently low, If EFA is to be achieved, a large extra number of children must attend school (Figure 8). In West Africa, increasing enrolment could result in some 21 million extra children attending primary school. If EFA is to be achieved, and PTRs are to be kept under 40, approximately 486,000 extra teachers must be working in 2015. This requires a 2.3 –fold increase on calculated current teacher recruitment rates. Required teacher recruitment is not much impacted by HIV because prevalence rates in West Africa are comparatively low in the continent.

By contrast, in Central Africa, if 100% net enrolment were to be achieved by 2015, there would be only 28% more children in schools than if enrolment continued at the 2008 level (the equivalent percentage in West Africa is 50%; see figure 8). However, pupil-teacher ratios in Central Africa are typically higher than those in West Africa due to lack of teachers, so many more teachers need to be recruited to achieve EFA. In fact, due especially to the contribution of a small number of countries whose PTRs are expected to exceed 100 by 2015 if rates are constant, i.e. Congo, CAR, Chad and Rwanda, the number of teachers required to achieve EFA in Central Africa must increase 2.3 – fold; and recruitment to achieve that, 5-fold. HIV has a non-negligible impact on required recruitment since prevalence is higher, but constitutes a much smaller proportion of required recruitment as the number of extra teachers required is greater than the starting number (most of the teachers required for EFA will need to be recruited, so HIV status of current teachers is less relevant). Likewise, teacher HIV prevention, VCT and treatment would have a minor impact on recruitment required, though the positive impact of healthy teachers on children's education always makes aiming to maximise teacher prevention and treatment worthwhile.

East Africa needs to reduce its PTRs, partly due to successes in increasing enrolment; Kenya, Tanzania and Uganda all have NERs of over 85% (Table S2). It is also the region with the second-highest HIV prevalence after Southern Africa. Therefore this region has much to gain from prevention and treatment of HIV and AIDS. Many teachers are required in order to achieve EFA, in part due to Ethiopia's teacher deficit, which is a consequence of a low NER, high PTR and large school-age population. The number of teachers is required to increase 2.2-fold, and teacher recruitment, 3.8-fold. Increasing ART to 80% of those requiring it would result in a 3% decrease in recruitment required to achieve EFA in Uganda, Kenya and Tanzania.

**Figure 9:**
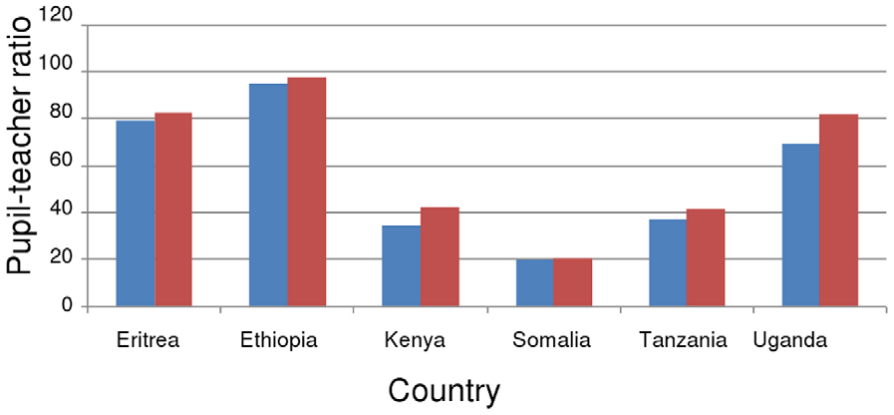
Impact of HIV on 2015 pupil-teacher ratio in East African countries. Red bars represent the most likely situation based on expected HIV impact; Blue bars represent 2015 PTR where the impact of HIV has been counterfactually removed in the model.

The potential impact of prevention on the pupil-teacher ratio is apparent from figure 9; in five East African countries, the expected 2015 PTR at current rates of recruitment is higher than if the HIV epidemic had not occurred. In Kenya and Tanzania, the epidemic may make the difference between achieving, or not achieving, Education For All.

**Figure 10:**
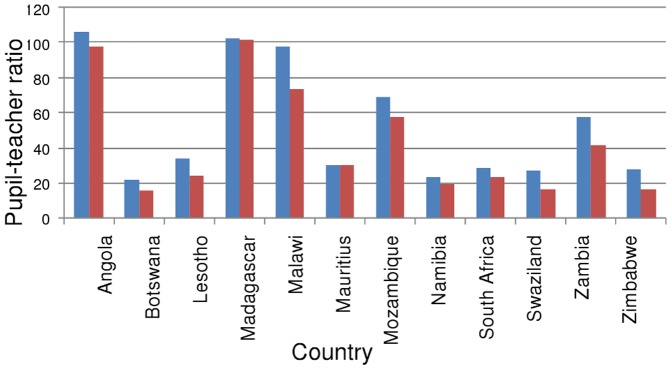
Impact of HIV on 2015 pupil-teacher ratio in Southern African countries. Red bars represent the most likely situation based on expected HIV impact; Blue bars represent 2015 PTR where the impact of HIV has been counterfactually removed in the model.

In Southern Africa, although HIV impacts are much larger, in many countries PTRs are already low, the exceptions being Angola, Malawi, Madagascar, Mozambique and Zambia (figure 10), and NERs are moderate. In this region, a moderate effort is required to achieve EFA (a 2.6-fold increase in teacher recruitment by 2015 to achieve a 1.6-fold increase in the number of teachers), and the large potential impact of HIV on this is due to the very high prevalence in these countries.

**Figure 11:**
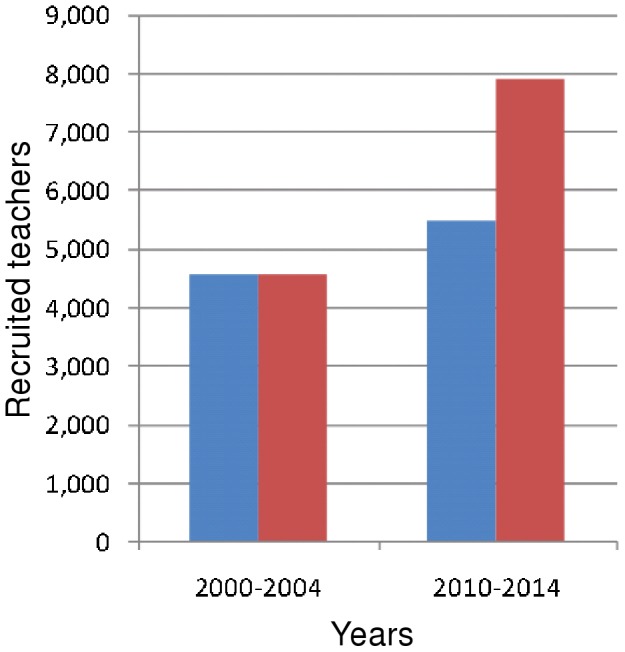
Impact of increased ART provision on recruitment required to achieve Education For All in Zambia. 2000–2004 = Estimated historical teacher recruitment in Zambia; 2010–2014 = estimated future teacher recruitment required to achieve Education For All, where future teacher ART roll-out is at current levels (red bars); and where it is increased to the maximum (blue bars).

Treating teachers in high-prevalence countries with relatively low current roll-out and a modest effort required to achieve EFA can result in many fewer new teachers being necessary. An example of such a country is Zambia, where 1.7-fold increases on 2000–2004 levels of teacher recruitment are required unless ART roll-out is completed for all teachers, in which case, only a 1.2-fold increase is sufficient (figure 11).

#### 5. Costs to the health and education sectors of the impact of HIV on education

We estimate that the cost of HIV to education is around $159 million 2009 USD, and the cost to the health system of VCT and ART to teachers to be $37 million 2009 USD (table 3). The cost is greatest in sub-Saharan Africa, followed by East Asia then the Caribbean, ordered in the same way as for non-cost impacts (figures 3–4). This is despite the unit costs tending to be greater in the Caribbean than in East Asia (Table S3).

**Table 3:** Summed costs of HIV to education and health in the three geographical regions, thousands of US$.

	Geographical Region	2008	2015 status quo	2015 universal access
Education	West Africa	$13,613	$13,979	$7,717
	Central Africa	$14,274	$12,621	$2,557
	East Africa	$10,417	$9,233	$5,855
	Southern Africa	$118,105	$110,033	$66,018
	sub-Saharan Africa	$156,409	$145,866	$82,147
	Caribbean	$764	$950	$724
	East Asia	$1,661	$1,903	$1,588
Health	West Africa	$14,556	$13,040	$27,336
	Central Africa	$4,096	$3,103	$7,485
	East Africa	$9,377	$8,639	$15,479
	Southern Africa	$3,579	$3,578	$8,602
	sub-Saharan Africa	$31,608	$28,360	$58,902
	Caribbean	$4,070	$3,270	$3,832
	East Asia	$1,361	$1,129	$1,529

Table 3 presents the likely change in costs between 2008 and 2015. In sub-Saharan Africa, where treatment and care remains at status quo, costs of HIV to education are likely to decline slightly due to discounting, even though impacts are likely to rise (figures 5–6). Treatment could greatly reduce this even more, to about half of current costs. Health costs are set to increase if treatment and care increases or remain similar otherwise.

In both the Caribbean and East Asia, education costs are projected to increase significantly unless universal access to ART is enabled, in which case they are expected to decline slightly. Health costs are expected to decrease where ART provision is merely maintained. This is due to the discounting of costs where the largest burden on health by far is VCT; in the higher prevalence areas, the increase in cost of ART increases sharply with increasing access as prevalence is set to rise. In the Caribbean, increasing access will only cause a small increase in health costs.

These results are not qualitatively different under the epidemic uncertainty scenarios.

#### 6. Costs and Benefits of Increased Access to ART

Table 4 presents the costs and benefits of increasing access to treatment. In sub-Saharan Africa, nearly 47,000 teachers' lives could be saved by facilitating access to treatment, along with over 50,000 teacher-years of absence, saving the education sector over $870,000 in teacher training and death benefits. Table 5 displays the costs per death averted and returns on investment into teacher health. The sub-Saharan African high returns are particularly pronounced in Southern Africa. This is because the high prevalence, coupled with the lower cost of drugs, makes mass-VCT, and treatment for all those found to require it, highly efficient.

**Table 4:** Costs and benefits of increasing ART and VCT in three global regions.

**Sub-Saharan Africa**		
Costs	Cost of increasing ART	$34,267
	Cost of increasing VCT	$184,803
Benefits	Teachers' deaths averted	46958
	Teacher-years of absenteeism saved	51515
	Total saving 2009–2015 to MoE of increasing ART and VCT use	$873,778
**Caribbean**		
Costs	Cost of increasing ART	$123
	Cost of increasing VCT	$2,909
Benefits	Teachers' deaths averted	100
	Teacher-years of absenteeism saved	220
	Total saving 2009–2015 to MoE of increasing ART and VCT use	$1,954
**East Asia**		
Costs		$246
	Cost of increasing VCT	$4,076
Benefits	Teachers' deaths averted	520
	Teacher-years of absenteeism saved	
	Total saving 2009–2015 to MoE of increasing ART and VCT use	$2,416

Costs are in thousands of US$.

## Discussion

We have found that teachers, a significant proportion of the workforce and on the front line as agents of future change, are impacted by HIV, in such a way as to require taking HIV into account when planning for EFA achievement. The potential disruptive effects of HIV on education provision are even broader than that estimated quantitatively, due to the powerful unquantified effects that teacher AIDS absences and deaths have on education provision; and that the economic effects of the impact of HIV are large but that the enhanced provision of ART to teachers is cost-saving.

We now discuss the sensitivity of the results to parameter variation; compare this model to another HIV impact model; contextualize our results into other estimates of the economic impact of HIV on education; consider the significance of our results for the Millennium Development Goals; and then summarize the principal results.

### Sensitivity and scenario analyses

1. **HIV epidemic scenario.** UNAIDS models output high, medium and low epidemic scenarios, which were input into Ed-SIDA to investigate scenario uncertainty in our results. The implications of this potentially large source of variation in model output have been presented throughout as figure error bars. This variability is related to uncertainty in HIV prevalence; in areas of lower prevalence, the percentage difference to outputs that epidemic scenario makes is higher. In high-prevalence Southern Africa, differences in epidemic scenario affect the outputs by up to around ±15%; and in low prevalence East Asia, an error of up to around ±50%. Nevertheless, when either the high and low epidemic scenarios are adopted, the qualitative conclusions reached by this analysis are not changed.
2. **Teacher Age.** We were able to source teacher age data for one-third of countries for which we provide impact, and used data from the nearest neighbor from which they were available for the others. As age distribution is a major predictor of HIV prevalence, it is important to examine errors arising from using a neighbor's data. Teacher age distribution may differ between countries due to societal changes. For example, the upheavals that Rwanda and Eritrea experienced in the early nineties means that their teachers, presumably like all their working population, were younger than in most countries; conversely in Kenya a policy of reduction in teacher recruitment led to an increase in the average age of teachers. We simulated the impact of possible errors due to application of another country's age profile by switching between countries' age distributions within regions while maintaining other parameters. This resulted in changes in prevalence of around 1–2%, resulting in changes of around ±20% in our model outputs; qualitative conclusions were not different to those found in the run presented in the main results.
3. **Teacher Relative Risk.** We have little information on whether being a teacher makes a person more or less at risk from HIV infection than a non-teacher of the same age and gender. There is some evidence that in some countries, (e.g. Rwanda), teachers are more at risk (unpublished analyses of DHS data by authors); and in others (e.g. South Africa), teachers are less at risk, by as much as 50% [16]. Our analyses of DHS data indicated that overall, there was no evidence to suggest that teachers had a different risk of infection than non-teachers, and the results presented here accordingly assume an equal risk. Varying relative risk in the bounds suggested by data had no impact on the principal qualitative conclusion that paying for ART & VCT in SSA provides a return.
4. **ART impact.** In this analysis we model ART as preventing a fixed proportion of deaths and absences, without the added complexity involved in explicitly modeling the stages of progression of the disease as, for example, the ASSA model does [17]. The mortality and morbidity rates of the treated population is expected to change over time due to the varying proportions of people in different stages of progression. The method we use represents true expected mortality rates better in the initial years after the initiation of mass ART, and this aspect of the method should be updated if the model is to be used after 2015. For the purposes of this analysis, the return on ART treatment in SSA Southern Africa is expected to hold true despite this effect.
5. **Combination of Impacts.** Singly, none of these potential sources of model error have the power to disrupt our conclusions, but if acting in concert, they might. Given that we do not know whether or why teachers might be per se more or less likely to be infected with HIV, then neither do we know whether an increase in susceptibility of teachers is associated with susceptible ages being more represented in the teacher population or a higher than average course of the epidemic. There was no association in our dataset between predisposition of teachers to HIV infection due to age-gender within a country and its overall HIV prevalence.

### Models used

UNAIDS models (EPP and spectrum) were used for HIV inputs as they are widely used and the methods are transparent and well-established. An alternative model of the impact of HIV on workforces is the ASSA model [17]. In general, potential advantages of the ASSA model over Ed-SIDA are its applicability to workforces of any sector or company and its more detailed treatment of these workforces, for example, stratification by band within a company workforce, and Ed SIDA's major advantage over ASSA is its facility to carry out multi-country/region analyses. ASSA's model uses different HIV impact inputs, but UNAIDS [19] found ASSA's population estimates consistent with those obtained from Spectrum which is used to provide HIV inputs to EDSIDA. Another potential disadvantage over Ed-SIDA is that it does not routinely output high and low HIV scenarios. The primary advantage of Ed-SIDA for this analysis is the ease with which it can combine projections across countries (the facility is also used to combine projections within countries [20]).).Spectrum has been criticized relative the ASSA HIV model for its lack of inclusion of reduced incidence following ARV treatment [21]. We model ART's impact as reducing a proportion of (1) deaths and (2) absenteeism; but as we consider teachers not to contribute to the dynamics of infection in the community, the contribution to decreased incidence is not included.

The education model results (e.g. teachers required to achieve EFA) are subject to less potential uncertainty, being predicated only on UIS data on teacher numbers and student enrolment, and attrition and recruitment data collected in-country.

### Impact on Teacher Numbers

This study shows the best estimates of the human and economic impact of HIV on education in the absence of comprehensive data on this impact. The stigma surrounding HIV makes objective data gathering difficult and by its nature, the extent of the information provided by model outputs cannot be gathered directly. The Universal Primary Education (UPE) goal [2] is hard to achieve in the short term in those countries which are furthest from attainment, due to low teacher numbers and high PTRs. Projections for high-PTR countries indicate that, quite independently of HIV, a major teacher shortage is looming, particularly in sub-Saharan Africa [22]. Much of Africa experienced a historical growth in teacher numbers between 1998 and 2002; however even if this were sustained, it would be insufficient for most countries to reach a PTR of 40∶1 by 2015, even with the current low student enrolment ratios. To achieve UPE, growth rates in teacher numbers above 9% per year would be needed in several countries, and in some countries greater than 20%; rates which are probably impossibly high.

Since the 1990s, it has been recognized that the HIV pandemic is likely to exacerbate the issue of teacher shortages, particularly for sub-Saharan Africa [23]. Early sectoral strategy and policy papers on HIV and education highlighted this issue [24], [25], but there has been some controversy about the impact of HIV and AIDS on education system staffing, reflecting different sources of data on teacher mortality [26], [27], [28]. Subsequent work has shown that AIDS mortality rates on the scale estimated here – less than 1.0% - can have significant consequences for teacher numbers [29], [30] and are in the range of empirical estimates [31]. Furthermore it is now recognized that successful efforts to provide universal access to care, treatment and support have significantly reduced teacher mortality in some counties, such as Botswana [32].

The debilitating illness that generally precedes death from AIDS implies loss of teacher contact time, quality, continuity and experience [33]. This has been recognized in previous analyses [34], [25], [29] and was shown by Grassly et al. [16] to be a potentially larger economic drain on the sector than the cost of recruitment and replacement of teachers resulting from AIDS-related mortality. The present analyses support this conclusion for all three regions examined. It is not known to what extent the impact of HIV and AIDS contributed to the high rates of absenteeism observed in the two most widely cited school surveys that examined teacher absenteeism [35], [36]. The high cost of absenteeism reflects the cost of paying a salary for both the absent teacher and for a substitute teacher. In the regions described here, substitution for absent teachers is commonly practiced in the Caribbean and South East Asia, but is uncommon in sub-Saharan Africa, where the most common result of an absent teacher is no teaching for that class. This obviously has major implications for education cost and quality, but these could not be explored here.

Estimated teacher HIV prevalence and mortality (table 1) are within the range described by other authors from data [33], [31].

The 2009 and 2010 EFA GMR [37], [38] estimate a rather lower level of required recruitment that we do: “If the world is to achieve UPE by 2015, it will need to recruit an estimated 18 million additional teachers. In sub-Saharan Africa, an additional 145,000 recruitments are needed annually – 77% above the observed increase between 1999 and 2006. South and West Asia will need an additional 3.6 million teachers.” [37] This 145,000 compares to our estimate for sub-Saharan Africa of additional recruitment of 347,000 (difference between baseline and required recruitment, table 2). It is not known how many years over which this annual recruitment was estimated; we estimate an increase during the years 2009–2014 inclusive, perhaps a shorter time than the previous GMR estimate. We estimate baseline recruitment from teacher numbers and attrition, which may differ from the methods used in the GMR. If these lower estimates are realistic, treatment facilitation will render a greater proportion of the required recruitment unnecessary.

### Epidemiology

The epidemiological results explored here point to the importance of care and support: the number of teachers living with HIV, absenteeism and deaths are all projected to increase in its absence. ART can reverse this upwards trend in absenteeism and death, but increases further the number of teachers living with HIV. For those teachers who are infected there is thankfully now increased access to care and support. Access is reportedly greatest in the Caribbean and lowest (and increasing rapidly) in SSA, although ART access may diminish in the wake of the financial crisis (e.g. [39], [40]). The estimates indicate that the number of teacher deaths, the mortality rate of teachers and the rate of teacher absenteeism in all three regions are lower now than in the mid 1990s. These large-scale trends do mask sub-regional differences; Southern Africa is projected to experience its greatest teacher mortality rates in the coming years.

The projections, which explore the epidemiological patterns to 2015, are based on two scenarios of access to ART. The first assumes that current levels of ART are not increased; treatment is maintained at the status quo. This implies: almost universal access in the Caribbean; somewhat less access in East Asia, with its small but growing epidemic; and low levels of access in sub-Saharan Africa combined with a still significant epidemic. The projections suggest that if there continues to be effective coverage with ART in the Caribbean there will be a sustained decline in the number of teacher deaths, the mortality rate of teachers and the rate of teacher absenteeism. For Africa and East Asia these measures are projected to remain at approximately current levels and may increase.

The second scenario assumes that universal access to care and support, including treatment, is achieved immediately. In all three regions this is projected to result in a significant and rapid decline in death, mortality and absenteeism. Note that both the underlying prevalence of HIV among teachers and the number of teachers infected will rise during this period while the impact is declining because it is the disease that is being managed and not the infection that is being prevented. Thus ART causes an increase in numbers of teachers potentially vulnerable to stigma and improvements in care and support systems may need to be instituted or improved to cope with the needs of this increasingly HIV positive population of teachers.

Resistance to first line therapy was not considered in this analysis because its effects are likely to be minimal in the years to 2015. Beyond this date it is likely to emerge first in the Caribbean and East Asia, where treatment scale-up has progressed fastest. Ed-SIDA contains functionality to include the impact of treating people with second-line therapy; and the impact of resistance and second-line therapy has been evaluated using Ed-SIDA in Trinidad and Tobago [20].

### Costs, and cost-benefit of treatment

The estimated costs in 2009 of providing VCT and ART at current coverage in the different regions (Table 3) suggest that the highest investment needed is in Africa, then South East Asia and then the Caribbean. If these costs are scaled per teacher (denominator is all teachers), they suggest a rather different picture, with both Southern Africa and Greater Mekong spending less than $10 per teacher and the Caribbean and other sub-Saharan African regions, between $10 and $20. This reflects both the greater costs in the Caribbean and the greater access. Note that the total costs scale with population for VCT, since all teachers are eligible for testing, but the cost of treatment scales with prevalence.

The benefits of treatment are highlighted in Table 4. The most stark benefit of spending on VCT and ART to 2015 is in saving teachers' lives; in sub-Saharan Africa, 46,958 deaths could be averted. There are potential deaths to be averted in all three areas, and there are teacher-years of absenteeism due to illness which will also be averted due to ART use. For members of the education sector, the financial gain to them by treatment (where VCT and ART costs are borne by the health sector) may also be a compelling argument for the increased roll out of ART and VCT to teachers, also detailed in table 4.

The results of the projections show that in all three regions the costs to the education sector are lower under scenario two, which assumes increased ART coverage. In both Africa and East Asia the reduction in additional costs of teacher supply attributed to HIV & AIDS is some 80% if there is universal access to VCT and ART. In the Caribbean the reduction is slightly less at 60%, largely because access is already high. These results suggest that in addition to the social and moral arguments, Ministries of Education have much to gain economically from encouraging their teachers to take full advantage of VCT and ART.

In the case of Africa, the high impact of HIV & AIDS and the relatively low baseline coverage of VCT and ART, conspire to make the interventions cost effective on the basis of the return to education alone. The projected cost of increased coverage between 2009–2015 is $219 million and this is estimated to result in a reduction in HIV-related teacher supply costs of $874 million. This suggests a return of 3.99 on the dollar, even ignoring the additional and important health and social returns. (Table 5)

**Table 5:** Costs per death averted and return on investment into VCT and ART between 2009–2015.

	sub-Saharan Africa	Southern Africa	Caribbean	East Asia
**Cost of ART and VCT per teacher's death averted**	$4,809	$1597	$30,320	$8,311
**Return on investment into ART and VCT to education sector**	$3.99	$23.08	$0.64	$0.54

In the Caribbean, current levels of coverage are already relatively high, and teachers relatively few and well supported with treatment. In this region the move from current coverage to universal coverage requires an investment of only $3 million. But because the impact of HIV & AIDS on supply is already partially addressed, the reduction in HIV-related education supply costs between 2009–2015 is estimated at only $2 million, suggesting an unfavorable return of 0.64 to the education sector (Table 5). It should be noted, however, that the saving to the education sector given universal access to ART and VCT is enough to fund provision of the required drugs to all teachers.

In East Asia, returns are low. The estimated HIV prevalence in teachers is low and ART coverage for those needing it likewise. By 2015 the estimated increase in prevalence among teachers is modest, but the cost of universal access will be relatively high at $4 million, largely due to the fixed cost of VCT for the almost one million teachers. The reduction on HIV-related supply costs are estimated at $2.4 million which, due to the high fixed costs to the health sector, translates into a return of only 0.56 to the education sector (Table 5).

The encouraging outcomes of scenario 2 are associated with significant costs to the health sector. It might be argued that these are sunk costs intended to save lives, reduce morbidity, and increase the quality of life, and that the benefits in terms of education are valuable additional consequences that may make teachers particularly important and cost-effective recipients for these health interventions.

The costs for which estimates can be made most readily are teacher training, absenteeism costs (salary) and ART. The cost of death benefits will probably vary greatly between countries depending on policy. Costs incurred by an in-service teacher death may be substantially more in countries which can afford greater payments. For example, in Guyana, the family of a teacher who has died receives at least one year's salary from the Ministry of Education on death, whereas many African countries have no policy of providing death benefit. Including the real costs incurred by Ministries of Education in countries would increase the cost-effectiveness of ART provision in the Caribbean and East Asia. The per-person cost of VCT is likely to decrease with the scale of VCT provision. Unit costs will probably decrease over time, thus making testing more cost-effective than it currently is. In addition to the relative cost-effectiveness gain due to increased death benefits in high-income countries, the Caribbean and East Asia are likely to scale- up VCT faster, which will also increase cost-effectiveness.

It is also important to note that the epidemiological and economic patterns described here do not assume any specific prevention intervention. The costs estimated here are those for managing rather than controlling the epidemic, and so the projected costs will continue in line with baseline prevalence projections, all else being equal.

As facilitating teachers' access to antiretroviral therapy may be cost-saving (table 5), ministries of education can choose to allocate resources to this. Unions can play a large role in advocating for VCT to be provided to all teachers, which occurred in, for example, Zambia.

With universal access, the numbers of teachers living with HIV is projected to increase. Universal access requires enhanced coordination between the health and education sectors, accompanied by an increasing role for support networks, including teacher unions, for HIV positive teachers.

There is a current increase in activity of HIV positive teachers' networks and associations (e.g. Kenepote in Kenya [41], and T'lipo in Malawi).The real number of teachers living with HIV and those requiring treatment is impossible to gauge empirically; such modelling approaches provide useful information for the functioning and advocacy of such groups. The collaboration of modellers with activists in this way will be useful for advancing the cause of these organisations.

Although not presented in this paper, unpublished estimates of the cost of providing necessary second-line therapy suggest that its addition will lead to a near-doubling of total costs of ART in 2015.

### Comparison of current with previous estimates

The 2002 estimates are around twice this estimate of $156 million for sub-Saharan Africa. This probably reflects three factors. First, the current estimates are based on several more years of understanding of the HIV epidemic, and of its impact on education in particular. For example, treatment roll-out has proceeded at an unprecedented pace in the past 5 years. Second, the new estimates are based on the UNAIDS revised estimates of prevalence, most of which were revised downwards significantly [42]. Finally, the epidemic peaked in most of these countries in the late 1990s, and estimates from the early part of this decade would have assumed a greater ongoing impact of HIV & AIDS than is actually now apparent.

Interestingly, neither of the 2002 estimates included any assumptions about testing or treating teachers. It is a testament to rapid progress made since then that current policy assumes that treatment access should be universal. For sub-Saharan Africa this policy adds an estimated $32 million to the 2008 costs, and the projections suggest that this cost will rise to some $59 million by 2015 if universal access is achieved.

### The Broader Context

This study focuses on the impact of HIV & AIDS on education supply specifically, and does not attempt to estimate all costs of the epidemic to the education sector. There are two main areas which are not examined here but would add significantly to the economic impact of HIV & AIDS on the education sector. The first of these is the need to provide specific support to orphans, children living with HIV [43] and other children made vulnerable by HIV & AIDS so that they can have access to schooling and remain in school long enough to complete an education. These costs may address tuition fees, local school levies and uniform purchase, but may also need to cover social support and basic living expenses. With the numbers of such children in sub-Saharan Africa estimated to be 50 million in 2010 [44] these costs are likely to be substantial. The second major cost omitted is the investment in prevention efforts by the education sector. The sector is a natural conduit for prevention efforts since it not only has the greatest direct, ongoing contact with children and youth in most countries, but also provides access to what is often more than 50% of the public sector workforce. Prevention requires costs for curricular efforts, such as appropriate training of teachers and provision of teaching materials, and for co-curricular efforts, such as peer counseling and peer education. While the curricular costs may be at the margins of education costs generally, provision of teaching materials and in-service teacher training may represent substantial additional investments.

The third MDG target of gender equality in education by 2005 was missed, and the target of gender equity by 2015 looks unlikely to be met on the current trajectory [42]. Gender is a critical element of both education and the HIV & AIDS response. In the present analyses gender is addressed specifically in terms of the input data, which are segregated to reflect the marked age- and gender-dependency of HIV infection. However, there are other almost certainly important gender-related factors that were not addressed through lack of data. For example, we do not know whether absenteeism is more likely to affect female teachers because of their care giving roles, nor whether care, support and treatment are more accessible to male teachers though evidence on this latter area is beginning to emerge [45]. Research in these areas would be of practical value since a more gendered analysis would allow for a more targeted response.

The key conclusions of these analyses are:

Our estimates of the cost of HIV for education supply in sub-Saharan Africa in 2008 are lower than the 2002 estimates, reflecting the lower prevalence of infection and a better understanding of the impact of HIV on the sector.

Our total costs due to HIV in the education sector are however greater than previous estimates if the costs of VCT and ART are included. The costs of ART and VCT were not estimated in the 2002 studies.

Universal access by teachers to VCT and ART is beneficial to education supply in all the three regions assessed.

In the Caribbean, the saving to the education sector of ART provision to all teachers requiring it could fund the drugs to treat them (but not universal VCT).

In sub-Saharan Africa, where the impact of HIV is greatest, the investment in universal access to both ART and VCT is cost-effective on the returns to education supply alone; investing in this care and support brings returns 3.99–fold higher than the investment.

## Materials and Methods

### Model description

Ed-SIDA models the processes which affect the number of primary school teachers in each country from before the start of the impact of the HIV epidemic in 1980 to the target date from the achievement of EFA in 2015. It evaluates the impact of HIV on these processes (i.e. teacher attrition), and also estimates the number of teachers living with HIV over time, and their absenteeism due to AIDS illness. It applies costs of deaths, absence, teacher training, VCT and ART to generate economic outputs. It combines an epidemiological model, using UNAIDS' EPP and Spectrum models [46] to project the course of the HIV epidemic, and an education planning model, to project teacher supply needs in the face of that epidemic. It outputs on each country each year and sums by region. Where input data are missing it looks up the nearest country with that data to allow the model to run. For a mathematical description of the epidemiological model; see Supplementary Information S1. The model used is [15], developed from [16] by allowing user exploration of future addition of treatment for teachers, and allowing the probability of death to depend on age, gender and expected time since infection.

Epidemiological models provide country-specific epidemiological projections of HIV prevalence, incidence and AIDS deaths. EPP and spectrum output these values for each gender and 5-year age group, and give high, low and medium estimates. They use inputs from antenatal (ANC) prevalence data, calibrated using national prevalence estimates, and fit a descriptive epidemic curve. Demographic data from each country is used to back-calculate HIV prevalence and AIDS mortality in each 5-year age-gender group. Uncertainty in the course of the HIV epidemic in each country due to varying the plausible model fits to ANC data gives the high, low and most plausible estimates, which are the basis on the uncertainly intervals around model results shown in this analysis.

The education model is a national planning tool, where education data were sourced directly from Ministries of Education and the UNESCO Institute of Statistics (UIS) online database at http://stats.uis.unesco.org, and other sources.

It is presumed that AIDS absenteeism does not displace significant amounts of non-AIDS absenteeism i.e. that expected AIDS absences are simply additional to measured background rates of absenteeism. Data from Namibia [46] imply that displacement reduces impacts due to absenteeism only by 1–5%.

### Model Processes

#### 1. Estimating the effort required to achieve EFA

We took into account two measures of EFA achievement: Net Enrolment Rate (NER) and Pupil-Teacher Ratio (PTR) and compared two achievement scenarios; status quo and full achievement. EFA achievement is defined as, by 2015, NER = 100%; and PTR is at most 40. NER is increased linearly by the model from 2008 levels to 100% in 2015. Pupil-teacher ratio is defined as the product of the school-age population and gross enrolment ratio (GER), divided by the number of teachers. GER for 2009 on is proportional to the NER, scaled on the average ratio of GER to NER. Teacher recruitment is defined as the number of new teachers appointed in one year, and in past years is calculated from the difference between one year's teachers and the subsequent year's teachers, and teacher attrition. Conversely, for future years, teacher numbers depend on previous year's teachers, teacher recruitment and teacher attrition. Under “Status quo” teacher recruitment is the average of the last 5 years' recruitment, whereas “EFA achieved” recruitment is that required to achieve a linear increase in the number of teachers such that EFA (PTR<40) is met in 2015.

If the expected pupil-teacher ratio in 2015 is less than the Fast Track Initiative's usual target ratio of 40∶1 where net enrolment rate is increased to 100%, then teacher recruitment would be expected to remain unaltered, and the model inputs “status quo” recruitment between 2009–2015. Most sub-Saharan African countries require increases in both enrolment and teacher recruitment to achieve EFA; whereas in the Caribbean and the Greater Mekong, although in some countries (especially Haiti), considerable effort is required in increasing enrolment, it is only in Cambodia that this needs to be accompanied by an increase in teacher recruitment if the countrywide target pupil-teacher ratio of 40 is to be met. Therefore the impact of HIV on EFA achievement is only examined in the SSA sub-regions.

#### 2. Deriving teacher HIV prevalence from population HIV prevalence

The population prevalence and mortality over time for each age and gender group thus obtained were combined with the age and gender profile of teachers in each country to predict teacher HIV prevalence and AIDS death rates respectively. Figure 1 shows examples of such data from Kenya. HIV infection status is highly dependent on age and gender (figure 1A). The age and gender distribution of teachers is unlike the pyramid distribution of a country with a growing population (figure 1B). Consequently, prevalence in teachers is likely to be very different from that in the general population.

In addition to the risks associated with age and gender, the teaching profession could confer either protection or additional risk of HIV infection [47]. The data to quantify the age and gender-stratified relative risk of HIV among teachers compared to the general population are sparse. The best assessment of that risk to date is that by [18], who sampled teachers from South African schools and found that teachers had a reduced risk of being infected. Lopman (pers comm.) also found teachers had a slightly lower rate of infection in rural Zimbabwe. Unpublished analyses of DHS data suggest that this risk varies between countries, and in some countries, teachers are more likely than non-teachers to be infected in their age and gender group, whereas overall, and in most individual countries, no significant difference can be detected (forthcoming publication). We therefore assume age and gender-stratified teacher risk of infection is the same as the general population.

#### 3. Modelling ART and VCT impact

ART is modelled as preventing a proportion of AIDS deaths and AIDS absenteeism in HIV positive people each year. The efficacy of ART was determined by comparing the mortality of people in low-income countries enrolled in ART programs [48] with untreated AIDS mortality (UNAIDS estimates). The proportion treated each year were taken from UNAIDS estimates (and future proportion treated was a model variable). To enable to calculation of the cost of VCT, it was assumed the proportion of those requiring treatment who were treated arose from the testing of that same proportion of people that year. i.e. treated/needing treatment = tested/teachers.

#### 4. Projecting the effect of different policy scenarios for the availability of ART

We examined two policy scenarios of the access of teachers to ART during the years 2009 until 2015. They are: 1) the status quo: the proportion of teachers having access to ART remains at 2008 levels until 2015; 2) immediate universal access: ART is made available immediately to all teachers who undergo annual VCT and are by this found to require and receive ART. It is assumed here that the proportion of HIV infected requiring ART is the same as in the general population, as estimated using UNAIDS models.

It should be noted that for countries in sub-Saharan Africa it is assumed that a maximum of 80% of teachers requiring ART can achieve access. Thus the estimated costs and benefits are lower than would be seen with the desirable but currently unrealistic assumption of universal health system access. In some countries, especially those in Asia and the Caribbean, access rates in access of 80% have been achieved, and for these countries the observed proportion was used as the upper bound for access..

#### 5. Estimating costs to the education and health sectors

Costs incorporated in the model are: 1) absenteeism, proportionate to salary payments for days not present due to AIDS illness; 2) death benefits payable per teacher AIDS death to the family of the deceased from the Ministry of Education (usually limited to funeral costs, and excluding pension); 3) the cost per AIDS death of training a teacher to fill the vacancy resulting from the loss; 4) the cost of voluntary counselling and testing (VCT) per year per eligible teacher; 5) provision of first-line ART therapy to teachers treated. There are important additional costs that could not be meaningfully estimated from available data on teachers, and so they have not been incorporated here. These include: treatment of opportunistic infections; transport to and from clinics; laboratory analyses to assess treatment eligibility; and patient support such as supplementary nutrition. The costs presented here, particularly those associated with the health sector, are therefore under-estimates.

### Model inputs

#### 1. Country-specific data and treatment of missing data, from 1980 to 2008

UNESCO Institute of Statistics and the Ministry of Education planning departments from individual countries were the primary sources for country-specific data. Teacher numbers were determined from a pre-epidemic start date of 1980 until 2008. Age and gender profiles for the teachers were presumed to be equal to those in 2008, or the latest available year. Where data were unavailable for one year, values were assumed to increase or decrease linearly between bounding years. Where data from 1980 or 2008 were unavailable, data were assumed to be equal between 1980 and the first year with an available data point, or between 2008 and the last year with a data point. Where no recruitment data and/or attrition data were available, the attrition was initially set at an estimated region-specific value (see Table S2), and the rate of recruitment was varied until the number of teachers calculated by the model equalled the reported number of teachers in that year. If there was a drop in teacher numbers which could not be accounted for by zero recruitment, the attrition rate was increased until the calculated and observed numbers of teachers were equal. The reported number of teachers was not therefore entered into the calculations directly, but was used to adjust attrition and recruitment rates so that the model matched the observation. Actual data entered from each country are listed in Table S2.

#### 2. Cost inputs

Education cost data (teacher salary, costs of absenteeism and death) were sourced directly from Ministries of Education, from country reports and from published sources. Where specific costs were unobtainable for individual countries, averaging of neighboring countries' costs was scaled on relative GDP per capita. Costs of treatment were obtained from WHO, and macroeconomic data from the World Bank. Costs were converted to 2009 dollars using a rate of inflation of 3%, and for future and past years were discounted at a rate of 3%. GDP was not converted using purchasing power parity to give the absolute, rather than relative cost. Table S3 shows the data from each country. Most untreated teachers living with HIV are expected to die within 10 years of graduating from teacher training college (unpublished analysis), so costs of training were not discounted by length of service.

## Supporting Information

Material S1 **Mathematical description of the model.**
(DOC)

Table S1 **List of countries for which estimations and projections were made, by region.**
(DOC)

Table S2 **Summary of data used in the model.** Data value followed by year in brackets. Where the data are age distributions, just the year is given. Age-gender disaggregated data were entered for HIV prevalence.
(DOC)

Table S3 **Principal costs entered into the model.**
(DOC)

## References

[1] UNESCO (2004) Education For All: The Quality Imperative (EFA Global Monitoring Report 2005). Paris: UNESCO

[2] Burnett N, et al. (2005). Literacy for Life. EFA Global Monitoring Report 2006. Paris, UNESCO.

[3] Caillods F, Kelly MJ, Tournier B (2008). HIV and AIDS: challenges and approaches within the education sector. IIEP brief for planners, Paris: IIEP-UNESCO 47p

[4] Jukes M, Simmons S, Bundy DAP (2008) “Education and Vulnerability: the role of schools in protecting young women and girls from HIV in southern Africa.”. AIDS 22 (4) S41–S46. 10.1097/01.aids.0000341776.71253.04 19033754

[5] UNESCO (2002) EFA Global Monitoring Report (2002): Education for All-Is the World on Track?, (Paris, UNESCO, 2002)

[6] Bruns B, Mingat M, and Rakotomalala R (2003) “Achieving Universal Primary Education by 2015: A Chance for Every Child,” Washington: World Bank.

[7] Makombe SD, Jahn A, Tweya H, Chuka S, Yu JK-L, et al (2007) A National Survey of Teachers on Antiretroviral Therapy in Malawi: Access, Retention in Therapy and Survival. PLoS ONE 2 (7) e620 doi:10.1371/journal.pone.0000620 17637836 10.1371/journal.pone.0000620 PMC1905945

[8] UNAIDS. (2008). Report on the global AIDS epidemic available at www.unaids.org/en/HIV_data (Accessed 2009)

[9] Iliffe J (2006). The history of the African aids epidemic. Athens, OH: Ohio University Press.

[10] Ghys PD, Walker N, McFarland W, Miller R, Garnett GP (2008) Improved data, methods and tools for the 2007 HIV and AIDS estimates and projections. Sex Transm Infect (Suppl 1) i1–4. 18647859 10.1136/sti.2008.032573 PMC2569833

[11] UNAIDS (2006). Report on the global AIDS epidemic available at www.unaids.org/en/HIV_data (accessed 2007)

[12] CAREC (2004). Status and trends: analysis of the Caribbean HIV/AIDS epidemic 1982–2002. Caribbean Epidemiology Centre. Accessible at http://carec.org/pdf/status_trends.pdf (accessed 2009)

[13] World Bank and Partnership for Child Development (2001). Modeling the Impact of HIV/AIDS on Education Systems: A training manual. The Ed-SIDA initiative. Washington DC: World Bank. First edition. Available at www.schoolsandhealth.org (Accessed 2009)

[14] World Bank and Partnership for Child Development (2006). Modeling the Impact of HIV/AIDS on Education Systems: A training manual. The Ed-SIDA initiative.Washington DC : World Bank. Second edition. www.schoolsandhealth.org (Accessed 2009)

[15] World Bank and Partnership for Child Development (2009). Modeling the Impact of HIV and AIDS on Education Systems: A training manual. The Ed-SIDA initiative.Washington DC : World Bank. Third edition. Available at www.schoolsandhealth.org (Accessed 2010)

[16] Grassly NC, Desai K, Pegurri E, Sikazwe A Malambo I, et al (2003) The economic impact of HIV/AIDS on the education sector in Zambia. AIDS 17: 1039–1044. 12700454 10.1097/00002030-200305020-00013

[17] Rosenberg S, Johnson L, Schneider D & Dorrington R (2000) ASSA multi-state select population model. Actuarial Society of South Africa, annual convention,

[18] Shisana O, Peltzer K, Zungu-Dirwayi N and Louw J (2005) the Health of Our Educators: A focus on HIV/AIDS in South African public schools Available at http://www.hsrcpress.ac.za/freedownload.asp?id=2082 (Accessed 2006)

[19] UNAIDS (2010) “Methods for estimating HIV incidence” UNAIDS quarterly update on HIV epidemiology/1Q 2010 Available at http://www.unaids.org/en/media/unaids/contentassets/documents/dataanalysis/epi_alert_2010Q1_en.pdf (acessed 2011)

[20] Navrozidis P, Risley CL & Beasley M (2011) Report on the situation analysis of HIV and AIDS in Trinidad and Tobago. London, PCD Available at www.schoolsandhealth.org (Accessed 2012)

[21] Nattrass N (2007) Modelling the relationship between antiretroviral treatment and HIV prevention: Limitations of the Spectrum AIDS Impact Model in a changing policy environment,. Afr J AIDS Res 6: 129–137. 25866062 10.2989/16085900709490407

[22] UNESCO UIS. (2006). Teachers and Education Quality: Monitoring Global Needs for 2015. UNESCO Institute for Statistics, Montreal.

[23] Kelly M (2000). Planning for Education in the Context of HIV/AIDS. Paris: IIEP-UNESCO.

[24] UNESCO (2002). Towards an African Response: UNESCO's Strategy for HIV/AIDS Education in Sub-Saharan Africa Dakar: UNESCO 86 p.

[25] World Bank (2002). Education and HIV/AIDS: A window of hope. Washington DC: World Bank. Available at www.schoolsandhealth.org (Accessed 2009)

[26] Bennell P, Hyde K, Swainson N (2002). The impact of the HIV/AIDS epidemic on the education sector in Sub-Saharan Africa: a synthesis of findings and recommendations of three country studies. Brighton: University of Sussex.

[27] Boler T (2003). Approaches to examining the impact of HIV/AIDS on teachers. Policy and Research Series/Save the Children and Action Aid International.

[28] Kinghorn and Kelly (2005). “‘The Impact of the Aids Epidemic’ Articles by Paul Bennell: Some Comments” The Journal of Development Studies, Vol. 41 , (No. 3): , pp.489–499

[29] Jukes MCH and Desai K (2005). Education and HIV/AIDS: Prevention, Treatment and Economic Impacts. A report prepared for the 2006 Education For All Global Monitoring Report, Paris, UNESCO.

[30] Carr-Hill R (2004). HIV/AIDS, poverty and educational statistics in Africa: evidence and indicators. UNESCO UIS. Montreal.

[31] Bennell P (2005), Countering the impact of the aids epidemic on the education sector In Swaziland. Knowledge and Skills for Development, Brighton.

[32] Bennell P (2006) Antiretroviral drugs are driving down teacher mortality in sub-Saharan Africa. An update on the data on teacher deaths in five high-prevalence countries. Knowledge and Skills for Development, Brighton

[33] Badcock-Walters P, Desmond C, Wilson D, Heard W (2003). Educator mortality in service in KwaZulu Natal: A consolidated study of HIV/AIDS impact and trends. Mobile Task Team on the Impact of HIV/AIDS on Education, Health Economics and HIV/AIDS Research Division, University of Natal.

[34] Grant KB, Gorgens M, and Kinghorn A (2004). Mitigating the Impact of HIV on Service Providers: What Has Been Attempted, What Is Working, What Has Not Worked, Where and Why? Study commissioned by the U.K. Department for International Development Service Delivery Team (Workstream on Capacity Development and Human Resources) in collaboration with the U.S. Agency for International Development. London.

[35] Schleicher A, Siniscalco M-T and Postlethwaite TN (1995). The Conditions of Primary Schools: A Pilot Study in the Least-developed Countries. A mimeographed report to UNESCO and UNICEF. Hamburg.

[36] Chaudhury N, Hammer J, Kremer M, Muralidharan K, and Rogers FH (2004). Teacher and Health Care Provider Absenteeism: A Multi-Country Study. World Bank.

[37] UNESCO (2008). EFA Global Monitoring Report 2009: Overcoming Inequality: Why Governance Matters. UNESCO: Paris.

[38] UNESCO (2009). EFA Global Monitoring Report 2010: Reaching the marginalized. UNESCO: Paris.

[39] Mills EJ, Ford N, Nabiryo C, Cooper C & Montaner J (2010) Ensuring sustainable antiretroviral provision during economic crises. AIDS 24 (3) 341–343. 20019576 10.1097/QAD.0b013e3283357e0f

[40] World Bank (2009). Averting a Human Crisis During the Global DownturnWashington DC : World Bank

[41] Bundy DAP, Aduda D, Woolnough A., Drake LJ, & Manda S. (2009). Courage and Hope: Stories from Teachers Living with HIV in sub-Saharan Africa. World Bank, Washington DC

[42] Brown T, Grassly NC, Garnett G, Stanecki K (2006) Improving projections at the country level: the UNAIDS Estimation and Projection Package 2005. SEX TRANSM INFECT 82: 34–40. 16735291 10.1136/sti.2006.020230 PMC2576727

[43] Cooper ES, Risley C, Drake LJ (2007) Bundy DAP (2007) HIV as part of the lives of children andyouth as life expectancy increases: Implications for education. Journal of International Cooperation inEducation 10 (1) 101–113.

[44] UNICEF 2005. The State of the World's Children, United Nations Children's Fund, New York (2004).

[45] Castro V, Duthilleul Y, Caillods F (2007). Teacher absences in an HIV and AIDS context: evidence from nine schools in Kavango and Caprivi (Namibia), UNESCO and IIEP

[46] UNAIDS 2009. EPP and spectrum models; Available at http://www.unaids.org/en/KnowledgeCentre/HIVData/Epidemiology/EPI_software2009.asp (Accessed 2010)

[47] Clarke DJ (2008). Heroes and villains: Teachers in the education response to HIV. Available at www.iiep.unesco.org (accessed 2009)

[48] The Antiretroviral Therapy in Lower Income Countries (ART-LINC) Collaboration, ART Cohort Collaboration (ART-CC) groups (2006) Mortality of HIV-1-infected patients in the first year of antiretroviral therapy: comparison between low-income and high-income countries The Lancet, Volume 367, Issue 9513, Pages 817–824, 10.1016/S0140-6736(06)68337-2 16530575

