# Supplementary material for: Economic Impact of HIV and Antiretroviral Therapy on Education Supply in High Prevalence Regions
Source: PLoS One. 2012 Nov 16;7(11):e42909. doi: 10.1371/journal.pone.0042909 (PMC3500246; doi:10.1371/journal.pone.0042909)
Supplement: Material S1 — Mathematical description of the model. (DOC) [file pone.0042909.s001.doc]

Supplementary material S1: Mathematical description of the model

$X_{g,a,c}(t)=$ number of HIV negative people in the population whose gender is *g* and whose age in years is *a* in country *c* at time *t*

$Y_{g,a,c}(t)=$ number of HIV positive people in the population who are of gender *g* and age *a* in country *c* at time *t*

For each country$N_{c}(t)=\sum_{g} \sum_{a} N_{g,a,c}(t)$

$X'_{g,a,c}(t)=$the number of HIV negative teachers of gender *g* and age *a* in country *c* at time *t*

$Y'_{g,a,c}(t)=$the number of HIV positive teachers of gender *g* and age *a* in country *c* at time *t*

$D_{g,a,c}(t)$ = AIDS deaths of gender *g* and age *a* in country *c* at time *t*

$R_{g,a,c}(t)$= number of teachers recruited of gender *g* and age *a* in country *c* at time *t*

$L_{g,a,c}(t)$ = teachers leaving teaching due to non-AIDS of gender *g* and age *a* in country *c* at time *t*

$A_{g,a,c}(t)$= teachers kept alive by ART of gender *g* and age *a* in country *c* at time *t*

We assume that teachers are recruited from the general population according to specific age-profile of recruitment and that the teacher population is small compared to the total adult population size ($X'_{g,a,c}(t)<<X_{g,a,c}(t)$ and $Y'_{g,a,c}(t)<<Y_{g,a,c}(t)$).

The dynamics of the teacher population can be given by the following difference equations using single year time steps[[1]](#footnote-2):

$$X'_{g,a,c}(t)=N'_{g,a,c}(t)-Y'_{g,a,c}(t)$$

$$Y'_{g,a,c}(t+1)=Y_{g,a,c}(t)/N_{g,a,c}(t)\cdot N'_{g,a,c}(t)\cdot\pi+A_{g,a,c}(t)$$

$$D_{g,a,c}(t+1)=Y'_{g,a}(t)\alpha_{g,a,c}(t)\cdot[(1-f(t))+f(t)(1-\varepsilon)]$$

$$R_{g,a,c}(t)=r_{g,a,c}[N'(t+1)_{c}-N'(t)_{c}-L(t)_{c}+D_{c}+O_{a=11,c}]$$

$$L_{g,a,c}(t+1)=l_{g,a,c}S(t)[N'_{g,a,c}(t+1)-D_{g,a,c}+O_{g,(a-1),c}-O_{g,a,c}]$$

$$A_{g,a,c}(t+1)=[Y'_{g,a,c}(t)A_{g,a,c}(t)/Y_{g,a,c}(t)-D-O_{g,a,c}+O_{g,a-1,c}][1-l_{g,a,c}S(t)]$$

Where

$N'_{g,a,c}(t)$= teachers of gender *g*, age class *a* and country *c* at time *t*. This is a data input (past) or calculated according to user-input assumptions (future – see Methods section)

$r_{g,a,c}$= proportion of recruitment that is of gender *g* and age *a* in country *c*

$f_{c}(t)$ = proportion teachers taking ART who need it in country *c*

$\varepsilon=$efficacy of ART

$S(t)$ = attrition rate scale parameter

$O_{g,a,c}(t)$= teachers aging from 5-year age class *a* to 5-year age class *a* + 1 of gender *g* in country *c* at time *t* (number aging determined by a linear spline of adjacent classes)

$\pi$ is the ratio of the prevalence in teachers to that in the general population (relative risk).

$\alpha_{g,a,c}(t)$= rate of AIDS mortality at time of gender *g* and age *a* in country *c* at time *t*

$l_{g,a,c}(t)$= rate of leaving the teaching profession due to non-AIDS death, retirement and loss to other professions by age, gender and country in gender *g* and age *a* in country *c* at time *t*

1. Analytical solutions to a continuous time equivalent to this model provide a slightly more exact way of modelling trends in the teachers population by ‘correcting’ for the fact that more than one event can be experienced by a teacher (e.g. joining the profession and getting HIV) in the same year. These solutions are available, but are deemed unnecessary in the current implementation given the inevitable error in the input data required. [↑](#footnote-ref-2)
