# Supplementary material for: Economic Impact of HIV and Antiretroviral Therapy on Education Supply in High Prevalence Regions
Source: PLoS One. 2012 Nov 16;7(11):e42909. doi: 10.1371/journal.pone.0042909 (PMC3500246; doi:10.1371/journal.pone.0042909)
Supplement: Table S1 — List of countries for which estimations and projections were made, by region. (DOC) [file pone.0042909.s002.doc]

Table S1, list of countries for which estimations and projections were made, by region.

| **ECCAS** | **ECOWAS** | **SADC** | **EAC/IGAD** |
| --- | --- | --- | --- |
| Burundi | Benin | Angola | Eritrea |
| Cameroon | Burkina Faso | Botswana | Ethiopia |
| Central African Republic | Cote d'Ivoire | Lesotho | Kenya |
| Chad | Gambia, the | Madagascar | Somalia |
| Congo | Ghana | Malawi | Tanzania |
| DRC | Guinea | Mauritius | Uganda |
| Gabon | Guinea-Bissau | Mozambique |  |
| Rwanda | Liberia | Namibia |  |
|  | Mali | South Africa |  |
|  | Niger | Swaziland |  |
|  | Nigeria | Zambia |  |
|  | Senegal | Zimbabwe |  |
|  | Sierra Leone |  |  |
|  | Togo |  |  |
|  |  |  |  |

| **Caribbean** | **East Asia** |  |
| --- | --- | --- |
| Bahamas | Cambodia |  |
| Barbados | Vietnam |  |
| Haiti | Laos |  |
| Jamaica | Thailand |  |
| Trinidad and Tobago | Myanmar |  |
| Belize |  |  |
| Guyana |  |  |
| Suriname |  |  |
|  |  |  |
