# Supplementary material for: Economic Impact of HIV and Antiretroviral Therapy on Education Supply in High Prevalence Regions
Source: PLoS One. 2012 Nov 16;7(11):e42909. doi: 10.1371/journal.pone.0042909 (PMC3500246; doi:10.1371/journal.pone.0042909)
Supplement: Table S2 — Summary of data used in the model. Data value followed by year in brackets. Where the data are age distributions, just the year is given. Age-gender disaggregated data were entered for HIV prevalence. (DOC) [file pone.0042909.s003.doc]

Table S2. Summary of data used in the model.

Data value followed by year in brackets. Where the data are age distributions, just the year is given. Age-gender disaggregated data were entered for HIV prevalence.

|  | **Education indicators** | | | | | | | | **HIV indicators** | | | |
| --- | --- | --- | --- | --- | --- | --- | --- | --- | --- | --- | --- | --- |
| **Country** | **Primary school teachers** | **Primary school teachers by age** | **Primary school teachers, %F** | **Recruitment by age and gender** | **Attrition rate** | **Attrition by age and gender** | **NER** | **GER** | **Relative risk of teacher infection** | **HIV prevalence (middle)** | **HIV prevalence (high)** | **HIV prevalence (low)** |
| Benin | 25,583 (2004) |  | 19% (2004) |  |  |  | 83% (2004) | 99% (2004) |  | 1.2% (2007) | 1.1% (2007) | 1.4% (2007) |
| Burkina Faso | 23,402 (2004) |  | 28% (2004) |  |  |  | 40% (2004) | 53% (2004) |  | 1.6% (2007) | 1.4% (2007) | 1.9% (2007) |
| Cote d'Ivoire | 48,308 (2003) |  | 24% (2003) |  |  |  | 56% (2003) | 72% (2003) |  | 3.9% (2007) | 3.2% (2007) | 4.5% (2007) |
| Gambia | 4,666 (2004) |  | 31% (2004) |  |  |  | 75% (2004) | 81% (2004) |  | 0.9% (2007) | 0.4% (2007) | 1.3% (2007) |
| Ghana | 88,461 (2006) | 2004 | 31% (2005) |  |  |  | 65% (2005) | 88% (2005) |  | 1.9% (2007) | 1.7% (2007) | 2.2% (2007) |
| Guinea | 25,361 (2004) |  | 24% (2004) |  |  |  | 64% (2004) | 79% (2004) |  | 1.6% (2007) | 1.3% (2007) | 2.2% (2007) |
| Guinea-Bissau | 3,518 (2001) |  | 20% (2001) |  |  |  | 45% (2001) | 70% (2001) |  | 1.8% (2007) | 1.3% (2007) | 2.6% (2007) |
| Liberia | 12,966 (2000) |  | 28% (2000) |  |  |  | 66% (2000) | 99% (2000) |  | 1.7% (2007) | 1.4% (2007) | 2% (2007) |
| Mali | 26,737 (2004) |  | 28% (2004) |  |  |  | 47% (2004) | 64% (2004) |  | 1.5% (2007) | 1.2% (2007) | 1.8% (2007) |
| Niger | 22,427 (2004) |  | 36% (2004) |  |  |  | 39% (2004) | 45% (2004) |  | 0.8% (2007) | 0.6% (2007) | 1.1% (2007) |
| Nigeria | 579,802 (2004) | 1990* | 51% (2004) | 2000* |  |  | 63% (2006) | 99% (2004) |  | 3.1% (2007) | 2.3% (2007) | 3.8% (2007) |
| Senegal | 32,005 (2004) |  | 24% (2004) |  |  |  | 66% (2004) | 76% (2004) |  | 1% (2007) | 0.7% (2007) | 1.4% (2007) |
| Sierra Leone | 17,327 (2004) |  | 30% (2004) |  |  |  | 52% (1997) | 145% (2004) |  | 1.7% (2007) | 1.3% (2007) | 2.4% (2007) |
| Togo | 22,210 (2004) |  | 13% (2004) |  |  |  | 79% (2004) | 101% (2004) |  | 3.3% (2007) | 2.7% (2007) | 4.1% (2007) |
| Cameroon | 55,266 (2004) |  | 40% (2004) |  |  |  | 70% (2000) | 117% (2004) |  | 5.1% (2007) | 3.9% (2007) | 6.2% (2007) |
| Central African Republic | 4,004 (1990) |  | 25% (1990) |  |  |  | 53% (1990) | 56% (2004) |  | 6.3% (2007) | 5.9% (2007) | 6.7% (2007) |
| Chad | 18,510 (2004) |  | 10% (2004) |  |  |  | 57% (2003) | 80% (2004) |  | 3.5% (2007) | 2.4% (2007) | 4.3% (2007) |
| Congo | 7,058 (2004) |  | 45% (2004) |  |  |  | 79% (1990) | 89% (2004) |  | 3.5% (2007) | 2.8% (2007) | 4.2% (2007) |
| Democratic Republic of the Congo | 162,797 (2003) |  | 26% (2003) |  |  |  | 61% (1994) | 62% (2003) |  |  | 1.2% (2007) | 1.5% (2007) |
| Gabon | 7,807 (2004) |  | 45% (2004) |  |  |  | 77% (2001) | 130% (2004) |  | 5.9% (2007) | 4.4% (2007) | 8.3% (2007) |
| Rwanda | 31,037 (2007) | 2002 | 53% (2007) |  |  |  | 94% (2007) | 147% (2007) |  | 2.8% (2007) | 2.4% (2007) | 3.2% (2007) |
| Angola | 32,144 (1998) |  | 24% (1998) |  |  |  | 53% (1998) | 64% (1999) |  | 2.1% (2007) | 1.7% (2007) | 2.5% (2007) |
| Botswana | 12,717 (2004) | 2004 | 79% (2004) | 2001 |  |  | 82% (2004) | 105% (2004) |  | 23.9% (2007) | 22.5% (2007) | 24.9% (2007) |
| Lesotho | 9,702 (2004) | 2001 | 80% (2004) |  | 5% (2001) |  | 86% (2004) | 131% (2004) |  | 23.2% (2007) | 21.9% (2007) | 24.5% (2007) |
| Madagascar | 64,270 (2004) |  | 60% (2004) |  |  |  | 89% (2004) | 134% (2004) |  | 0.1% (2007) | <0.1% (2007) | 0.2% (2007) |
| Malawi | 40,587 (2004) | 2004 | 46% (2004) | 2000 | 0% (2000) | 1995 | 87% (2007) | 116% (2007) |  | 11.9% (2007) | 11% (2007) | 12.9% (2007) |
| Mauritius | 5,322 (2005) | 2001 | 63% (2005) |  |  |  | 95% (2005) | 102% (2005) |  | 1.7% (2007) | 1% (2007) | 3.6% (2007) |
| Mozambique | 54,721 (2004) | 2001 | 30% (2004) | 2001 |  |  | 71% (2004) | 95% (2004) |  | 12.5% (2007) | 10.9% (2007) | 14.7% (2007) |
| Namibia | 14,442 (2003) | 2001 | 61% (2003) | 2001 |  |  | 74% (2003) | 101% (2003) |  | 15.3% (2007) | 12.4% (2007) | 18.1% (2007) |
| South Africa | 220,950 (2003) | 2004 | 74% (2003) | 2001 |  |  | 89% (2003) | 105% (2003) | 56% (2004) | 18.1% (2007) | 15.4% (2007) | 20.9% (2007) |
| Swaziland | 6,680 (2003) | 2001 | 75% (2003) | 2001 |  |  | 77% (2003) | 101% (2003) |  | 26.1% (2007) | 25.1% (2007) | 27.1% (2007) |
| Zambia | 46,414 (2004) | 2001 | 48% (2004) | 2001 |  |  | 80% (2004) | 99% (2004) |  | 15.2% (2007) | 14.3% (2007) | 16.4% (2007) |
| Zimbabwe | 61,251 (2003) |  | 51% (2003) | 2001 |  |  | 82% (2003) | 96% (2003) |  | 15.3% (2007) | 14.6% (2007) | 16.1% (2007) |
| Burundi | 24,452 (2006) |  | 55% (2006) |  |  |  | 75% (2006) | 103% (2006) |  | 2% (2007) | 1.3% (2007) | 2.5% (2007) |
| Eritrea | 7,942 (2005) | 1995* | 40% (2005) |  |  |  | 46% (2005) | 64% (2005) |  | 1.3% (2007) | 0.8% (2007) | 2% (2007) |
| Ethiopia | 121,452 (2006) |  | 45% (2005) |  |  |  | 56% (2005) | 93% (2005) |  | 2.1% (2007) | 1.8% (2007) | 2.2% (2007) |
| Kenya | 149,893 (2004) | 2005* | 44% (2004) | 2005* | 3%* (2015) |  | 75% (2006) | 111% (2004) |  |  | 7.1% (2007) | 8.5% (2007) |
| Somalia | 10,338 (1985) |  | 45% (1985) |  |  |  | 8% (1990) | 10% (1996) |  | 0.5% (2007) | 0.3% (2007) | 1% (2007) |
| Sudan | 110,323 (2004) |  | 66% (2004) |  |  |  | 43% (2000) | 60% (2004) |  | 1.4% (2007) | 1% (2007) | 2% (2007) |
| United republic of Tanzania | 151,882 (2006) | 2001 | 48% (2005) | 2001 |  |  | 91% (2005) | 106% (2005) |  | 6.2% (2007) | 5.8% (2007) | 6.6% (2007) |
| Uganda | 150,135 (2006) | 2001 | 39% (2006) |  | 5% (1998) |  | 92% (2006) | 114% (2006) |  | 5.4% (2007) | 5% (2007) | 6.1% (2007) |
| Cambodia | 48,736 (2007) |  | 43% (2007) |  |  |  | 89% (2007) | 119% (2007) |  | 0.8% (2007) | 0.7% (2007) | 0.9% (2007) |
| Lao People's Democratic Republic | 28,741 (2006) |  | 46% (2006) |  | 7% (2002) |  | 84% (2006) | 116% (2006) |  | 0.2% (2007) | 0.1% (2007) | 0.4% (2007) |
| Myanmar | 166,195 (2006) |  | 82% (2006) |  |  |  | 85% (2003) | 114% (2006) |  | 0.7% (2007) | 0.4% (2007) | 1.1% (2007) |
| Thailand | 321,930 (2007) |  | 60% (2007) |  |  |  | 94% (2007) | 106% (2007) |  | 1.4% (2007) | 0.9% (2007) | 2.1% (2007) |
| Viet nam | 353,608 (2006) |  | 78% (2006) |  |  |  | 93% (2002) | 100% (2003) |  | 0.5% (2007) | 0.3% (2007) | 0.9% (2007) |
| Bahamas | 1,756 (2005) |  | 88% (2005) |  |  |  | 91% (2005) | 101% (2005) |  | 3% (2007) | 1.9% (2007) | 4.2% (2007) |
| Barbados | 1,377 (2005) |  | 78% (2005) |  |  |  | 98% (2005) | 108% (2005) |  | 1.2% (2007) | 0.8% (2007) | 1.7% (2007) |
| Belize | 2,157 (2005) |  | 72% (2005) |  |  |  | 94% (2005) | 127% (2005) |  | 2.1% (2007) | 1.2% (2007) | 3.1% (2007) |
| Guyana | 4,185 (2005) | 2005* | 86% (2005) | 2005* | 11%* (2005) | 2003* | 89% (1993) | 132% (2005) |  | 2.5% (2007) | 1.4% (2007) | 3.7% (2007) |
| Haiti | 46,827 (2005) |  | 33% (1996) |  |  |  | 31% (2000) | 54% (2000) |  | 2.2% (2007) | 1.9% (2007) | 2.5% (2007) |
| Jamaica | 12,087 (2005) | 2003 | 89% (2005) |  |  |  | 90% (2005) | 95% (2005) |  | 1.6% (2007) | 1.1% (2007) | 2.1% (2007) |
| Suriname | 3,361 (2005) |  | 92% (2005) |  |  |  | 94% (2005) | 120% (2005) |  | 2.4% (2007) | 1.5% (2007) | 4.3% (2007) |
| Trinidad and Tobago | 7,863 (2005) |  | 72% (2005) | 1998 | 3% (1999) |  | 90% (2005) | 100% (2005) |  | 1.5% (2007) | 1% (2007) | 2.1% (2007) |

*New data presented here for the first time (to our knowledge).
