# Supplementary material for: Economic Impact of HIV and Antiretroviral Therapy on Education Supply in High Prevalence Regions
Source: PLoS One. 2012 Nov 16;7(11):e42909. doi: 10.1371/journal.pone.0042909 (PMC3500246; doi:10.1371/journal.pone.0042909)
Supplement: Table S3 — Principal costs entered into the model. (DOC) [file pone.0042909.s004.doc]

Table S3: Principal costs entered into the model:

| **Region** | **Country/countries** | **Item** | **Cost/cost range (US)** | **year** | **Source** |
| --- | --- | --- | --- | --- | --- |
| West Africa | All | ART | 159 | 2008-2009 | WHO [S1] |
| Central Africa | All | ART | 130 | " | " |
| East Africa | All | ART | 142 | " | " |
| Southern Africa | All except Mauritius | ART | 139 | " | " |
| Caribbean | All except Trinidad & Tobago & Barbados | ART | 192 | " | " |
| East Asia | All | ART | 111 | " | " |
| Caribbean | Guyana | VCT | 15 | 2006 | Ministry of Education* |
| East Asia | Thailand & Cambodia | VCT | 6-30 | 2002, 2006 | Web source |
| Sub-Saharan Africa | Tanzania, Sudan, Kenya, Uganda | VCT | 14-29 | 1997-2002 | Web source |
| Caribbean | Guyana | Salary | 775-1371 | 1998-2001 | Ministry of Education* |
|  | Jamaica | Salary | 7953 | 2003-2006 | Ministry of Education and youth* |
|  | Trinidad & Tobago | Salary | 16197 | 2008 | Ministry of Education* |
| East Asia | Cambodia, Viet Nam, Lao PDR | Salary | 180-480 | 2001-2002 | UNESCO |
| East Asia | Thailand | Salary | 5230 | 2005 | Thailand National Statistical Office, first quarter, 2005 |
| Sub-Saharan Africa | Many | Salary | 333-13,9000 | 1990-1996 | UIS (uis.unesco.org) |
| Caribbean | Guyana | Teacher Training | 1475 | 2006 | Guyana TTI* |
| Caribbean | Trinidad and Tobago | Teacher Training | 11000 | 2003 | [S2] |
| East Asia | Viet Nam | Teacher Training | 250 | 2001 | [S3] |
| Sub-Saharan Africa | Ghana, Tanzania, Mozambique, Zambia, Eritrea*, | Teacher Training | 311-2031 | 1999-2003 | Ministry of Education and MoE reports |
| Caribbean | Guyana | Death Benefit | 1728 | 2001 | Ministry of Education* |
| East Asia | Thailand | Death Benefit | 263 | 2006 | Tsunami government funeral assistance |
| Sub-Saharan Africa | Zambia, Swaziland, Ghana, Uganda, Zambia, Kenya* | Death Benefit | 176-3873 | 2000-2004 | Ministry of Education |

*New data presented here for the first time (to our knowledge).

[S1] World Health Organization. (2008) Transaction Prices for Antiretroviral Medicines and HIV Diagnostics from 2004 to September 2008. A summary report from the Global Price Reporting Mechanism. Geneva, Switzerland. <http://www.who.int/hiv/amds/gprm/en/> (accessed Dec 2008)

[S2] Lewin KM, Keller C and Taylor E (2000, revised 2002). Teacher education in Trinidad and Tobago: Costs, financing and future policy. Multi-Site Teacher Education Research Project (MUSTER) Discussion Paper No. 9. Brighton, UK: Centre for International Education, University of Sussex.

[S3] Can NB, Long VV, Tam PT and Sinh NT (2001) Educational financing and budgeting in Viet Nam. Working document in the series: Financial Management of Education Systems, International Institute for Education Planning, UNESCO, Paris
